# Sleep spindles comprise a subset of a broader class of electroencephalogram events

**DOI:** 10.1093/sleep/zsab099

**Published:** 2021-04-15

**Authors:** Tanya Dimitrov, Mingjian He, Robert Stickgold, Michael J Prerau

**Affiliations:** 1 Division of Sleep and Circadian Disorders, Brigham and Women’s Hospital Department of Medicine, Boston, MA; 2 Harvard-MIT Health Sciences and Technology, Massachusetts Institute of Technology, Cambridge, MA; 3 Department of Anesthesia, Critical Care and Pain Medicine, Massachusetts General Hospital, Harvard Medical School, Boston, MA; 4 Department of Psychiatry, Beth Israel Deaconess Medical Center, Boston, MA; 5 Department of Psychiatry, Harvard Medical School, Boston, MA

**Keywords:** sleep spindles, multitaper spectral estimation, EEG, memory consolidation, NREM

## Abstract

**Study Objectives:**

Sleep spindles are defined based on expert observations of waveform features in the electroencephalogram (EEG) traces. This is a potentially limiting characterization, as transient oscillatory bursts like spindles are easily obscured in the time domain by higher amplitude activity at other frequencies or by noise. It is therefore highly plausible that many relevant events are missed by current approaches based on traditionally defined spindles. Given their oscillatory structure, we reexamine spindle activity from first principles, using time-frequency activity in comparison to scored spindles.

**Methods:**

Using multitaper spectral analysis, we observe clear time-frequency peaks in the sigma (10–16 Hz) range (TFσ peaks). While nearly every scored spindle coincides with a TFσ peak, numerous similar TFσ peaks remain undetected. We therefore perform statistical analyses of spindles and TFσ peaks using manual and automated detection methods, comparing event cooccurrence, morphological similarities, and night-to-night consistency across multiple datasets.

**Results:**

On average, TFσ peaks have more than three times the rate of spindles (mean rate: 9.8 vs. 3.1 events/minute). Moreover, spindles subsample the most prominent TFσ peaks with otherwise identical spectral morphology. We further demonstrate that detected TFσ peaks have stronger night-to-night rate stability (ρ = 0.98) than spindles (ρ = 0.67), while covarying with spindle rates across subjects (ρ = 0.72).

**Conclusions:**

These results provide compelling evidence that traditionally defined spindles constitute a subset of a more generalized class of EEG events. TFσ peaks are therefore a more complete representation of the underlying phenomenon, providing a more consistent and robust basis for future experiments and analyses.

**Statement of Significance**

In this paper, we demonstrate that the current definition of spindle activity, which is based on historical observations electroencephalogram (EEG) waveforms, greatly undersamples from the oscillatory events underlying the phenomenon. This study is the first to systematically reexamine spindle activity from first principles using time-frequency events as the basis of observation, employing both manual and automatic detection methods. In doing so, we show that TFσ peaks, time-frequency peaks in the 10–16 Hz range, provide a more comprehensive, robust, and stable characterization of the spindle phenomenon. The high intra-individual stability and increased statistical power of TFσ peaks will likely enhance future studies associating spindle activity with memory, aging, and psychiatric and neurodegenerative disorders, as well as with large-scale epidemiological and genetic studies.

## Introduction

From its inception in the 1920s until the late 1960s, the electroencephalogram (EEG) was almost exclusively presented as brainwave traces drawn by mechanical pen on paper [1–4]. Consequently, much of the way that brain state is currently characterized has its historical basis in those features of the EEG time domain trace easily observable by eye. In particular, sleep research heavily relies on the visual inspection of the EEG time trace within a polysomnogram (PSG). Current clinical standards [5], which have remained virtually unchanged from the late 1960s [6], divide sleep into four stages: rapid eye movement (REM) and non-REM (NREM) Stages 1–3 (N1–N3). The different stages are defined by oscillatory activity across a set of canonical frequency ranges as well as specific waveform patterns.

One of the most prominent waveform patterns observed in the sleep EEG is the spindle, originally observed as waxing-waning 14 Hz oscillatory bursts [2]. The presence of trains of spindle waveforms in the sleep EEG chiefly defines N2 sleep [6], during which spindles have been observed to occur at an average rate of ~2–3 events/minute [7, 8]. Spindles have garnered substantial attention through numerous studies linking spindle activity to memory consolidation and neural plasticity during sleep [9, 10], as well as recent studies associating deviations in spindle activity and morphology with aging [11], Alzheimer’s disease [12], epilepsy [13], schizophrenia [14], and autism [15].

Spindles were first discovered in 1935 by Loomis, Harvey and Hobart through visual inspection of the EEG time domain traces on paper [2]. Nearly a century later, visual inspection of spindles by expert scorers is still the de facto “gold standard” in sleep research. The American Academy of Sleep Medicine (AASM) defines spindles as “A train of distinct waves with a frequency of 11–16 Hz (most commonly 12–14 Hz) with a duration of >0.5 second, usually maximal in amplitude using central derivations” [5]. Given this broad definition, there is often large variability in spindle counts reported between different human scorers on the same data set [16]. While numerous quantitative methods have been developed to automatically detect spindles [17], these approaches typically use hand-scored spindles as the standard by which performance is measured and parameters are tuned. Therefore, these automated methods ultimately serve to replicate imperfect human scoring rather than to identify objective markers of the neurophysiological phenomenon underlying spindles.

The fundamental problem in visual identification of spindles is that transient oscillatory signals are exceptionally difficult to parse by eye. This is because the sleep EEG generally involves the superposition of many components across a wide range of frequencies. Thus, a strong low-frequency activity during NREM sleep could completely obscure a small amplitude signal at another frequency. This inherent obfuscation raises the question of whether only those spindles of the highest amplitude occurring during times of quiescent low-frequency activity are being detected, thus potentially subsampling from a larger class of event. If this is indeed the case, current understanding of spindles might be biased by these historical observations, which would implicitly undersample spindle activity. Consequently, numerous reservations about human scoring of spindles have been voiced [16, 18–21], advocating for more objective EEG analyses in clinical sleep medicine [22, 23].

Spectral analysis, which decomposes a signal into its different frequency components [24] has been a long-standing tool for EEG analysis [25]. Recent work has illustrated how aspects of the dynamic oscillatory structure of the sleep EEG, including spindles, not visible in the time domain, can be readily observed in the time-frequency domain visualized by the multitaper spectrogram [26]. Any transient oscillatory activity, by definition, will appear as a salient time-frequency peak on the spectrogram. Thus, the phenomenology of spindles might be better characterized through the lens of time-frequency analysis, which disambiguates the dynamics of simultaneously occurring time-varying oscillatory activity.

There have been several studies using time-frequency analysis for spindle detection and exploration of oscillatory phenomena [27–32]. It is also common to analyze traditionally detected time domain spindles in the time-frequency domain. However, to our knowledge, no study has systematically characterized spindle activity from first principles using time-frequency phenomenology as the basis of observation with direct comparison to traditionally scored spindles. This line of inquiry is crucial to revealing any limitations inherent in the current time-domain definition of spindles, allowing us to move towards a more objective and evidence-based understanding of the underlying activity. In this study, we examine spindle activity with both hand-scoring and automated detection methods in both time and time-frequency domains using multiple datasets. We provide a comprehensive characterization of the properties of traditionally defined spindle waveforms in comparison to time-frequency activity observed in the sigma (10–16 Hz) range.

## Methods

### Datasets

We examined sleep recordings from three independent datasets: (1) patients in the DREAMS Sleep Spindle Database [33], (2) healthy control subjects in a previously published study [34], and (3) healthy young subjects in a high-density EEG study [35]. Details of each dataset are described below.

The DREAMS Sleep Spindle Database [33] is a public dataset consisted of 30 minutes excerpts taken from clinical PSG recordings in eight patients (age range: 31–53, sex: 4F/4M) with different sleep disorders (dyssomnia, restless legs syndrome, insomnia, apnea/hypopnea syndrome). Two trained experts blinded to the underlying sleep stages manually scored spindles on 30-second epochs according to the Rechtschaffen and Kales criteria [6]. One expert scored the full record for six patients; however, the other expert incompletely scored the records. We analyzed excerpts from the six patients with the complete expert scoring (2 recordings from C3-A1, one at 50 Hz and one at 100 Hz sampling rates; 4 recordings from CZ-A1 at 200 Hz sampling rate). We used this dataset to compare hand-scoring of events in the time and time-frequency domains.

The control subjects from Wamsley et al. [34] included 17 healthy participants (age range: 26–45, sex: 3F/14M), screened to ensure no history of mental illness, family history of schizophrenia spectrum disorder, or psychoactive medication use. Two full-night PSG recordings were digitally acquired from each participant at a sampling rate of 100 Hz using an Embla N7000 system (Medcare Systems, Buffalo, New York). The recording montage included five to seven EEG channels (F3, F4, C3, C4, Pz, O1, O2) referenced to the linked mastoids. Data from electrode C3 were used for all analyses in the present study. A random subset (six subjects) of the second-night recordings was drawn to extend hand-scoring in the time-frequency domain to the full-night length. We analyzed all recordings with automated detection methods in both time and time-frequency domains.

**Figure 1.**
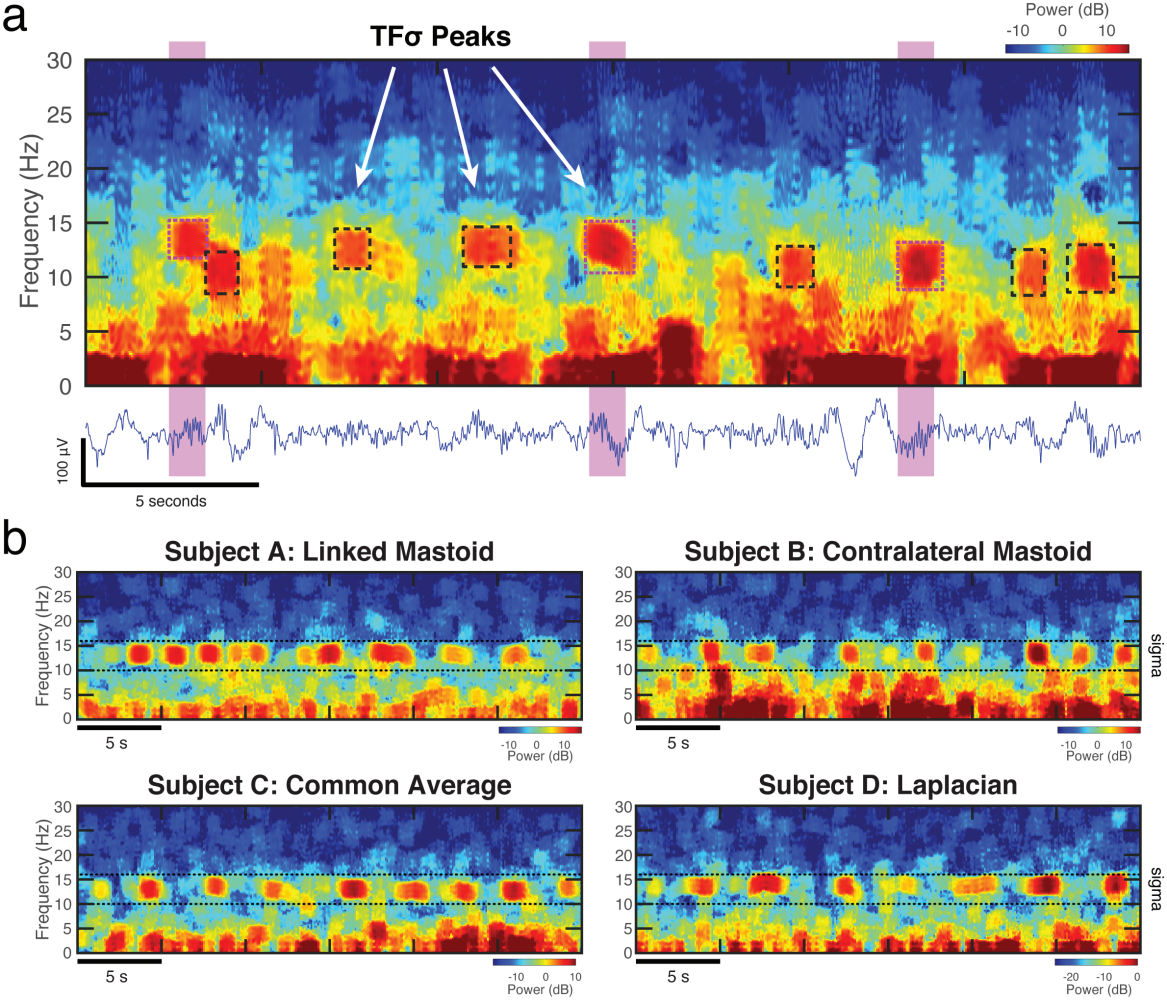
Spindles appear to be a subset of a broader class of time-frequency peaks in the sigma range of the spectrogram (TFσ peaks). (a) Salient peaks were identified by hand-scoring in the time-frequency domain (dashed boxes). Traditionally scored time domain spindles (highlighted regions) directly align with a subset of TFσ peaks (magenta dashed boxes) in the spectrogram of a 30-second segment of C3 data during N2 sleep. (b) The observations of numerous TFσ peaks appear to be ubiquitous and are seen across several datasets and referencing schemes, as illustrated by 30-second N2 epochs from four different subjects. The sigma range (10–16 Hz) is demarcated by horizontal dashed lines.

The data from Prerau et al. [35] contained a cohort of ten healthy right-handed subjects (age range: 19–32, sex: 5F/5M) with BMI <30, screened for sleep disorders and medication use. Full-night PSG were acquired at a sampling rate of 500 Hz with a 64-channel Brain Vision EEG cap. The data were downsampled to 200 Hz and offline rereferenced to common average and Laplacian referencing schemes. We analyzed the central channel 4 as the proxy for C3 in the standard clinical montage. Two recordings from this dataset were presented as examples of the abundant spindle-range activity on spectrograms in Figure 1. No spindle analysis was performed, as detailed discussion of referencing is out of the scope of the present study.

### PSG data processing

We preprocessed recordings prior to hand-scoring in the time-frequency domain and applying automated detection algorithms. For recordings from the DREAMS Sleep Spindle Database, we omitted sleep staging and artifact rejection to best match the expert scoring procedure that was blinded to sleep stages and automatically flagged artifacts. The standard protocol for visual sleep stage scoring was followed by trained polysomnography technologists and standardized to AASM guidelines [5]. For details, see the associated studies [34, 35].

Artifact rejection was implemented with a custom MATLAB function performing an iterative detection of artifacts based on z-scored signal amplitudes. To detect high-frequency noise, raw EEG traces were first filtered to above 35 Hz with an 8th order IIR high pass filter. Hilbert transform was then applied to the filtered signal. The amplitudes of the instantaneous signal were converted to a logarithmic scale, smoothed with a 2-second running average window, and spline detrended with 300 knots. The resultant signal was z-transformed over time, and time points with absolute z-scores above 4 SDs were marked as artifacts. This procedure was repeated after each iteration by recomputing z-scores until no time point deviated from 0 by more than 4 SDs. To detect artifacts with broadband energy, the original EEG traces were filtered to above 2 Hz with an 8th order IIR high pass filter. The same steps as described for high-frequency noise were followed, and time points beyond 4 SDs were iteratively marked as artifacts until convergence. Time points detected to contain either high-frequency noise or broadband noise (or both) were considered artifacts and removed from subsequent analyses.

EEG spectrograms were computed using the multitaper method [24, 26, 36] with the following parameters: 1 second window length, 0.05 second step size, time-half-bandwidth product (TW) of 2, 3 Slepian tapers, 2^10^ minimum number of discrete Fourier transform points (NFFT), and constant detrending within each window. For detailed explanations of the multitaper parameters for sleep analyses, see our previous publication [26] and tutorials on http://www.sleepEEG.org/multitaper.

### Extraction of time-frequency event properties

In this study, we compare the morphological properties of the EEG activity underlying events detected by different methods. One challenge for this analysis is that can often be difficult to reconcile property values from methods that use different assumptions or operate on different data transformations. For example, it is not meaningful to compare event duration or magnitude in time versus time-frequency methods, as the values are derived from separate domains and are not actually the same property. Furthermore, for hand-scored events, properties are highly contingent on crude manually selected regions in software (e.g. drawing boxes around events) or imposed fixed criteria (e.g. the fixed 1 second event duration in DREAMS hand-scoring). To reconcile these differences, we developed a generic time-frequency procedure to extract properties of the most salient transient EEG event occurring during a given detected time periods. The goal of this approach is to provide a unified framework for property estimation and comparison across detection procedures.

Our approach uses the time-frequency domain as the basis for event property estimation, since transient oscillatory activity will appear as salient time-frequency peaks in the spectrogram. This algorithm extracts time-frequency local maxima from the spectrogram by looking for regions with well-defined peak-like structure in both time and frequency dimensions. This is done in two steps, leveraging the concept of peak prominence, which describes the height of a peak relative to its local baseline. The first step is a “frequency step,” which detects peaks in the EEG power spectrum at each time and estimates the prominence of the largest peak in the spindle frequency range. The second step is a “time step,” which detects temporal peaks in the time trace of the prominence values obtained from the “frequency step.” Each temporal peak identified in the “time step” therefore corresponds to a well-defined local maximum on the spectrogram. To describe the morphology of these time-frequency peaks, we compute the following properties: prominence, duration, central frequency, and bandwidth.

We then use these identified peaks and properties for comparison between different hand or automated spindle detection methods. For a given detected event (from any method), we identify the most prominent peak occurring during that time period and assign the properties of that peak to that event. In doing so, we can use a common framework for event property comparison regardless of how the event was detected. Details of the prominence algorithm and property extraction are described in the Supplementary Material.

### Hand-scored and automated event detection

In the present study, we investigated “spindle-like” activity with both hand-scoring and automated detection methods in both time and time-frequency domains. To avoid confusion, we have introduced the following terminology used throughout the paper: *Sleep spindles* are reserved for the traditionally defined EEG waveform events in the time domain, typically following the AASM manual description as distinct waves with frequency range 11–16 Hz and lasting at least 0.5 seconds [5]. A *time-frequency peak* (TF peak) is used to describe a distinct region of enhanced activity in the time-frequency domain, observed as a local maximum in the spectrogram. While TF peaks can theoretically occur at any frequency, here we operationally define *sigma-range time-frequency peaks* (TFσ peaks) to refer to salient time-frequency peaks representing transient activity in the spindle frequency range (which we define as 10–16 Hz to keep consistent with the Wamsley detector described below), which can be easily visualized on spectrograms.

#### Hand-scoring of spindles

Expert hand-scoring is provided as part of the DREAMS Sleep Spindle Database. We used the scoring results from the second expert to identify spindles in the time domain on the first six excerpts. The expert scoring marked 1-second-long intervals that were subjectively judged to contain spindles. Per the convention of clinical sleep spindle scoring, time traces with hand-scored spindles are displayed after filtering to 0.3–35 Hz. This filter is only applied for visualization.

#### Automated detection of spindles

Recently, Warby et al. examined six published sleep spindle detection algorithms and found the Wamsley spindle detector [34] to best agree with the hand-scoring gold standard [17]. Based on this result, we employed the Wamsley detector as implemented in Warby et al. for automated detection of spindles in the time domain. The Wamsley detector transforms a single-channel EEG signal using an 8-parameter complex Morlet wavelet with scale parameters corresponding to the 10–16 Hz frequency range. The wavelet transform signal is then smoothed with a 100 ms moving average window. A detection threshold is set to be 4.5 times the mean of a wavelet magnitude statistic, the square of the real component of the squared wavelet coefficient, during N2 sleep. A sleep spindle event is detected when the wavelet signal exceeds this threshold for at least 0.3 second.

The Warby implementation of the Wamsley detector differs from the original Wamsley implementation by adding an upper limit of spindle duration at 3 seconds and imposing a 1-second minimal separation between two consecutive spindle onset times. We found these two changes to have negligible impacts on detection results. We kept them in the Wamsley detector to follow the exact same algorithm provided by Warby et al. It should be noted that spindle events specified by the Wamsley detector (10–16 Hz, durations of 0.3–3 seconds) differ slightly from the AASM definition of spindles as 11–16 Hz waves with durations above 0.5 second. Nevertheless, we still use *spindles* to refer to these detected spindle events, as the algorithm was developed to mimic and validated by hand-scoring of spindles in the time domain [17, 34].

#### Hand-scoring of TFσ peaks

Salient TFσ peaks were visually identified from the multitaper spectrograms on 30-second epochs by a trained scorer (T.D.) and manually marked with enclosing boxes using a customized MATLAB toolbox, which we have made publicly available. As TFσ peaks appear on the spectrograms as regions with increased activity, hand-scoring of TFσ peaks followed a set of rules: an event was visually scored as a TFσ peak if it was distinguishable from the background activity as a salient region of increased spectral power with sharp boundaries, well-contained along both time and frequency dimensions, and if the region bounds fell within the 9–17 Hz frequency range. We add ±1 Hz to the range to conservatively account for the main lobe bandwidth of the TFσ peak in the time-frequency domain. After scoring, we used the detected central oscillation frequency of each event (computed using the property extraction method described above), to restrict analysis to only those events falling within 10–16 Hz. No specific cutoff was adopted for event durations.

#### Automated detection of TF peaks

We developed a data-driven clustering method to automatically identify TFσ peaks in the time-frequency domain based on prominence values of local maxima on the multitaper spectrograms. There are abundant local maxima in the spindle frequency range throughout the night. Yet spindles and more generally spindle-like activity should be (1) well-formed TF peaks (2) robustly prominent relative to the background activity. Together, these factors help separate identifiable TFσ peaks from noise. We therefore looked at the prominence of all detected well-formed TF peaks extracted using the property extraction algorithm outlined above. The peak prominence quantifies the extent to which a local maximum stands out from the background activity, giving a numerical value for the distinctiveness of a peak in the time-frequency domain. We converted the prominence values of all local maxima to the logarithmic scale due to observed log-normal distributions. In order to derive a principled separation of events from noise unique to each individual, we applied a two-class k-means clustering algorithm [37] on the prominence values for each subject. The cluster with higher mean prominence was labeled as TFσ peaks of interest, and the other cluster with lower prominence was labeled as noise peaks. This choice was motivated by the observation of bimodal or skewed peaks within the prominence distributions. Prior to k-means clustering, we excluded peaks with durations shorter than 0.3 second to match consensus [17]. We further excluded peaks with frequency bandwidths less than half of the spectral resolution of the multitaper spectrograms (4 Hz/2 = 2 Hz), as peaks of this bandwidth are not resolvable by the spectral estimator. After k-means clustering, we exclude any detected events outside the 10–16 Hz range, to match with the method described in Wamsley et al.

#### F1-optimization of spindle detector thresholds

The Wamsley detection threshold at 4.5 times the mean coefficient magnitude is the default value optimized to match hand-scored time-domain spindles. To assess the performance of the Wamsley detector with varying thresholds, we reduced the threshold scalar from 4.5 in order to relax the rarity assumption on event rates. For the six DREAMS control subjects with hand-scoring in the time-frequency domain, we optimized the Wamsley threshold for the best agreement with hand-scored TFσ peaks based on F1 scores. We elaborate on the details of F1 computation below. To determine the F1-optimized threshold for each subject, we varied the Wamsley threshold from 0.01 to 4.5 in steps of 0.01 and selected the threshold producing the maximal F1 score. This produced individualized F1-optimized thresholds for each subject. We also computed a single group F1-optimized threshold that maximized the mean F1 across all subjects (Supplementary Material).

### Statistical analysis

Having detected spindles and TFσ peaks, we aimed to compare them systematically through various metrics. Here we describe all analyses conducted to compare detected events in the order of increasing levels of processing and use of statistical tests. The different analysis techniques complement each other and address distinct aspects of detected events. Taken together, these analyses provide a comprehensive comparison of spindles in the time domain and TFσ peaks in the time-frequency domain.

#### Event selection

With the exception of the DREAMS data, which was blinded to sleep staging, all analysis was confined to events occurring NREM Stage 2 (N2) sleep, in order to match the design specifications of the Wamsley detector, which is optimized for N2 analyses. Events within 3 seconds of a detected artifact or non-N2 stage were removed from analysis to avoid partial events due to clipping. Events detected with central frequency falling outside the 10–16 Hz range were removed from analysis to facilitate direct comparisons across the time and time-frequency domains.

#### Aggregate event spectrograms

To compute the aggregate event spectrograms, events were aligned based on the troughs of the filtered time-domain signals. For each detected event, the central trough time was defined as the minimum of the 10–16 Hz bandpassed time-domain signal during the event duration. For each event, the segment ±1.5 seconds around the trough was extracted from the multitaper spectrogram. Aggregate event spectrograms were computed using the element-wise median of all events across subjects, converted to the dB scale for visualization.

#### Confusion matrix statistics

Detection of spindle-like activity is a classification problem, of which confusion matrices can be used to assess detection performance of different methods [17]. We treated hand-scoring of TFσ peaks as the “ground truth” for defining true positive and false positive events. However, we do not assume hand-scored TFσ peaks as the best representation of the true underlying neurophysiological activity. Rather, we constructed the confusion matrices this way because TFσ peak hand-scoring can be used as the invariant method across comparisons (Tables 1–3). Using this fixed “ground truth” facilitates comparing spindle-like activity across time and time-frequency domains by providing a common reference for confusion matrix statistics. Specifically, we define true positive, false positive, and false negative events as:

**Table 1.** DREAMS Sleep Spindle Database: hand-scored TFσ peaks and spindles

	TFσ peak hand	Spindle hand				
Subject	Rate (events/minute)	Rate (events/minute)	Precision	Recall	F1	Overlap %
1	9.5	4.6	0.89	0.43	0.58	73
2	7.0	2.0	0.85	0.26	0.40	70
3	4.5	1.6	0.89	0.32	0.47	68
4	8.5	1.2	0.64	0.09	0.16	68
5	7.4	3.2	0.90	0.39	0.54	80
6	6.4	3.2	0.92	0.48	0.63	77
Mean ± SD	7.2 ± 1.7	2.6 ± 1.3	0.85 ± 0.10	0.33 ± 0.14	0.46 ± 0.17	73 ± 5.1

This table shows the event detection comparisons of time-domain hand-scored spindles and TFσ peaks in the segments of DREAMS Sleep Spindle Database. TFσ peaks have about 2–3 times of the event rates (number of hand-scored events per minute during NREM2 Stage 2 sleep) of sleep spindles. Confusion matrix statistics (described in *Methods*) show that the vast majority of sleep spindle events are also detected as TFσ peaks, as reflected by the high precision scores. However, the reverse is not true as the low recall scores indicate only about one-third of TFσ peaks are scored as sleep spindles. F1 scores, which give a more balanced measure of the agreement between the two methods, indicate a moderate match between the two class of hand-scored events. Nevertheless, when an EEG event is jointly detected both as a TFσ peak and a sleep spindle, there is substantial overlap between the two methods.

**Table 2.** Event rates in the six subjects analyzed for comparisons of automated methods

	TFσ peak hand	Spindle auto	Spindle auto F1-optimized	TFσ peak auto
Subject	Rate (events/minute)	Rate (events/minute)	Rate (events/minute)	Rate (events/minute)
1	8.4	3.6	8.6	11.5
2	10.9	3.1	10.2	11.5
3	12.2	3.7	12.0	11.6
4	9.2	2.9	8.5	10.4
5	8.9	3.4	9.1	10.4
6	12.7	3.8	12.9	11.8
Mean ± SD	10.4 ± 1.8	3.4 ± 0.3	10.2 ± 1.9	11.2 ± 0.6

This table shows comparisons of event rates (number of hand-scored events per minute during NREM2 Stage 2 sleep) across various spindle detection methods. These methods are analyzed in six subjects from the control subject cohort with full-night recordings and hand-scored TFσ peaks and different from the six segments in DREAMS database shown in Table 1. Auto-detected spindles, similar to hand-scored spindles, have one-third of the rate of hand-scored TFσ peaks. F1-optimized thresholds were selected individually for each subject based on the threshold scalar value producing the maximal F1 score when comparing against hand-scored TFσ peaks. As expected, both auto-detected spindles with optimized thresholds and automated detection of TFσ peaks achieve similar rates to those of hand-scored TFσ peaks.

**Table 3.** Confusion matrix statistics in the six subjects analyzed for comparisons of automated methods

A. Spindle Auto					
Subject	Precision	Recall	F1	Overlap %	
1	0.86	0.37	0.52	49	
2	0.99	0.28	0.44	50	
3	0.99	0.30	0.47	36	
4	0.98	0.33	0.49	39	
5	0.96	0.37	0.53	39	
6	0.97	0.30	0.46	36	
Mean ± SD	0.96 ± 0.05	0.33 ± 0.04	0.48 ± 0.04	41 ± 6.3	
B. Spindle Auto F1-optimized					
Subject	Precision	Recall	Max F1	Overlap %	Threshold at Max
1	0.74	0.76	0.75	62	0.93
2	0.85	0.79	0.82	61	0.45
3	0.91	0.90	0.90	56	0.19
4	0.85	0.82	0.83	48	0.67
5	0.87	0.88	0.88	58	0.56
6	0.86	0.89	0.88	57	0.24
Mean ± SD	0.85 ± 0.06	0.84 ± 0.06	0.84 ± 0.05	57 ± 5.1	0.51 ± 0.28
C. TFσ peak Auto					
Subject	Precision	Recall	F1	Overlap%	
1	0.68	0.95	0.79	76	
2	0.86	0.92	0.89	74	
3	0.93	0.91	0.92	62	
4	0.79	0.95	0.86	73	
5	0.81	0.96	0.88	73	
6	0.90	0.88	0.89	64	
Mean ± SD	0.83 ± 0.09	0.93 ± 0.03	0.87 ± 0.04	70 ± 5.8	

This table presents the confusion matrix statistics (described in Methods) when comparing various automated detection methods against hand-scored TFσ peaks. These methods are analyzed in six subjects from the control subject cohort with full-night recordings and hand-scored TFσ peaks and different from the six segments in DREAMS database shown in Table 1. (A) Results for auto-detected spindles recapitulate the patterns observed in Table 1 of hand-scored spindles, except precisions scores are now close to 1 due to more reliable scoring of spindles by the automated algorithm. (B) Automated spindle detection using F1-optimized thresholds show both high precision and recall scores, as the optimization goal was to maximize the F1 scores. However, varying thresholds are needed to achieve maximal F1 score in different subjects, highlighting the challenge of pre-selecting a single uniform threshold for all subjects. (C) The automated detection algorithm of TFσ peaks also achieves high precision and recall scores in comparison to hand-scored TFσ peaks. Importantly, the F1-scores are numerically higher in all subjects relative to the best possible F1-score using spindle detection algorithms with adjusted thresholds. This result demonstrates the benefit of detecting TFσ peaks directly from the time-frequency domain and the robustness of the automated detection algorithm to emulate the process of hand-scoring TFσ peaks.


$$\begin{array}{ll} true\;positive\;(TP)=\; & an\;event\;hand\text{-}scored\;as\;a\;TF\sigma \;peak\;and\;\\ & also\;detected\;by\;the\;other\;method \end{array}$$

$$\begin{array}{ll} false\;positive\;(FP)=\; & an\;event\;not\;hand\text{-}scored\;as\;a\;TF\sigma \;peak\;but\;\\ & detected\;by\;the\;other\;method \end{array}$$

$$\begin{array}{ll} false\;negative\;(FN)=\; & an\;event\;hand\text{-}scored\;as\;a\;TF\sigma \;peak\;but\;\\ & missed\;by\;the\;other\;method \end{array}$$

Confusion matrix statistics (precision, recall, F1) were calculated individually for each subject (Tables 1 and 3). We define *precision* and *recall* as:


$$\begin{array}{ll} precision=TP/(TP+FP)=\; & fraction\;of\;events\;detected\;by\;the\;other\;\\ & method\;hand\text{-}scored\;as\;TF\sigma \;peaks \end{array}$$

$$\begin{array}{ll} recall=TP/(TP+FN)=\; & fraction\;of\;hand\text{-}scored\;TF\sigma \;peaks\;\\ & detected\;by\;the\;other\;method \end{array}$$

F1 score [38] takes the harmonic mean of precision and recall, and it gives a more balanced measure of the agreement with TFσ peak hand-scoring and is often used in the absence of the ability to define a true negative:


$$\begin{array}{l} F1=2\times precision\times recall/(\;precision+recall) \end{array}$$

For all confusion matrices, we used an event-based analysis to determine TPs. When a hand-scored TFσ peak overlapped in time with an event detected by the other method, it was treated as a TP event without a minimal overlap requirement. In rare cases, multiple events detected by the other method overlapped in time with a single TFσ peak marked by hand-scoring. Then multiple TP events were counted. On the contrary, if a single event overlapped with multiple TFσ peaks, it was counted as one TP event.

To complement this event-based analysis without an overlap threshold, we measured the extent of overlap during TP events. An overlap percentage is defined as the fraction of intersection over union, i.e. the percentage of the number of sample points jointly marked by hand-scoring of TFσ peaks and the other method over the total number of sample points detected by either or both methods. This overlap percentage was calculated separately for each TP event, and the average percentage across TP events is reported for each subject (Tables 1 and 3). It should be noted that this metric uses the event time as defined by the detection methods and does not use the procedure for general event property extraction defined above.

#### Statistical tests on event properties

We first conducted paired-sample *t*-tests on the property medians of detected events. For any two methods being compared, we computed the median values for each subject of the four properties (prominence, duration, central frequency, and bandwidth), respectively. As results of different detection methods are inherently related to the same subject-specific neurophysiological activity, paired-sample *t*-tests assess the systematic differences on properties of detected events. Median is chosen over mean due to observed tailed distributions on all properties and being less sensitive to rare outlier events.

A consistent pattern observed throughout the analyses is that there are many more TFσ peaks detected in the time-frequency domain than spindles detected in the time domain. We therefore would like to understand the regions of different properties on which more TFσ peaks were detected than spindles. Practical challenges associated with this question arise from (1) individual-subject differences on event properties and (2) skewed distributions of properties of events detected by time and time-frequency methods. To address these issues, we conducted statistical tests on individually z-scored and binned properties.

**Figure 2.**
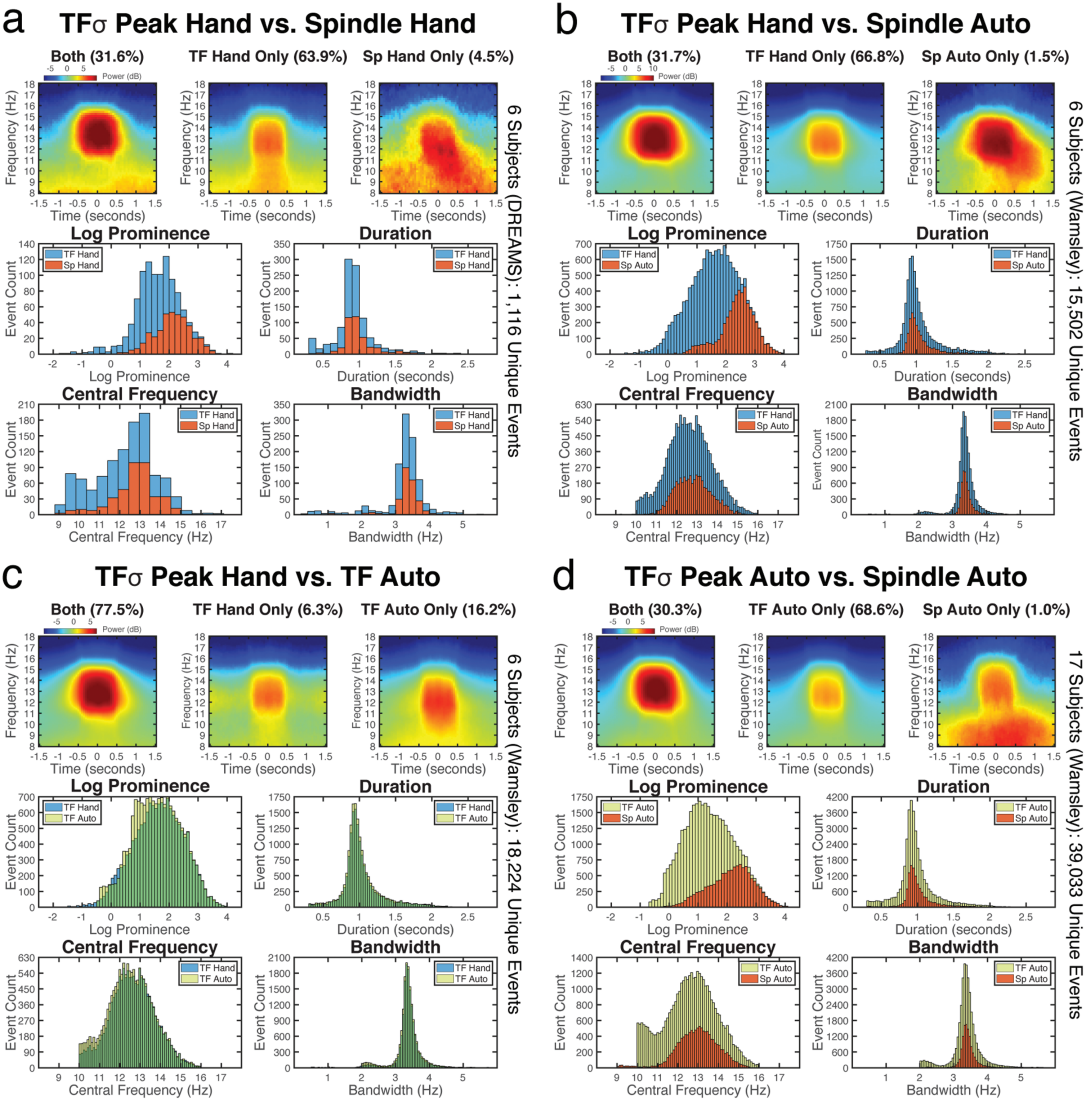
Spindles are a morphologically similar subset of TFσ peaks. We compare the average event spectrograms and morphological property distributions of spindles (“Sp”) and TFσ peaks (“TF”) detected by hand-scoring (“Hand”) and auto-detection (“Auto”). In (a)–(d), the top row shows the average spectrograms from all unique events detected by both methods (“Both”) or uniquely identified by a given method (“Only”). The bottom four subpanels show the histograms of event counts for the given methods corresponding to different morphological properties: prominence, duration, central frequency, and bandwidth. The results in (a), (b), and (d) show that the morphological properties of spindles nest within those of TFσ peaks, and that spindle detection has an implicit bias towards sampling TFσ peaks with higher prominence. Thus, spindles do not exhibit characteristics consistent with a class of EEG events distinct from TFσ peaks. The results in (c) show that TFσ peaks identified by the auto-detection method agrees strongly with hand-scored TFσ peaks.

For each subject, we computed the median and SD of a property based on detected spindle events. We then performed a modified z-score (for outlier robustness) on the properties of both TFσ peaks and spindles (Z = (X-median)/SD). When comparing hand-scoring and automated detection of TFσ peaks, we z-scored (modified) based on the auto-detected events. In all cases, we then divided the z-scored property values into seven bins. The bins have width of 1 SD and the center values of the middle five bins are –2, –1, 0, 1, –2 SD. The first bin ends at –2.5 SD and is extended to include negative infinity. Similarly, the last bin starts at 2.5 SD and is extended to include positive infinity. The counts of events falling in the seven bins were calculated for both methods being compared and divided by the total N2 sleep duration to obtain rate measures for each subject. A Wilcoxon signed-rank test was then applied across subjects to compare the rates of TFσ peaks with the rates of spindles in each bin. We used the nonparametric signed-rank test over paired t-tests due to the lack of normality in event rates (unlike the median statistic that can be assumed to be normally distributed). Two-sided statistics were used to examine the seven bins of each property in all comparisons shown in Figure 2. We show the z-scored event property histograms as well as bin-wise test boxplots in the Supplementary Material. In summary, these bin-wise tests provide details on the separation of distributions observed on property histograms (also see Supplementary Material for Kolmogorov-Smirnov distributional tests). The results of median-based and bin-wise statistical testing together help describe the phenomenology of TFσ peaks, in reference to spindle properties already known and described in the time domain.

#### Correlation of event rates and night-to-night consistency analysis

We conducted correlative and linear regression analyses on the automated detection results in the time and time-frequency domains for control subjects from the Wamsley study [34]. Full-night recordings from two consecutive nights were analyzed for all 17 control subjects. We computed the correlation coefficient (ρ) between the rates (events/minute during N2 sleep) of spindles detected by the Wamsley detector and the rates of auto-detected TFσ peaks, as well as of event rates across the two consecutive nights for both auto-detected TFσ peaks and spindles. We also fitted linear regressions to all correlations using the robust least-squares fitting method with bisquared weights. Robust linear regression was used over standard least-squares to mitigate the influence of potential outliers. We report the fitted coefficients and 95% confidence bounds for the regressions.

To explicitly test whether there is greater intra-individual stability of TFσ peaks than spindles, we conducted a permutation test on the difference in Pearson correlation coefficients. Specifically, the event rates were first z-scored within each method, and the cross-night correlation coefficients were computed. The difference in correlation coefficients for the two methods provided an observed correlation difference. We then randomly permuted the method tag of all z-scored event rates and recomputed the correlation difference for the shuffled methods. This process was repeated 10 000 times to form a null distribution of correlation difference. The probability of the null distribution exceeding the observed difference was taken as the permutation-corrected probability for the observed correlation difference being statistically significant.

## Results

Following the current clinical definition [5], we henceforth use the term *spindles* to refer to the class of time-domain EEG waveform events identified by or verified against expert hand-scoring. We use *time-frequency peaks* (TF peaks) to refer to the class of salient peaks (local maxima) in the spectrogram, typical of the time-frequency transform of a transient oscillatory waveform. It should be noted that we use both terms to refer exclusively to waveform events within the EEG signal, with no presumption of the underlying physiological origin. Given the plurality of definitions of the sigma/spindle range [39], we use 10–16 Hz for maximum agreement between the manual and automated methods analyzed herein.

### Spindles appear to be a subset of a broader class of time-frequency events

In order to first probe the underlying time-frequency phenomenology, we followed the footsteps of Loomis, Harvey, and Hobart, but this time in terms of activity within the sleep EEG spectrogram. Figure 1a shows a typical 30-second epoch of N2 sleep recorded from a central electrode (C3) from the DREAMS Sleep Spindle database, along with expert hand-scored spindles highlighted on the corresponding time trace. The spindles directly correspond to well-circumscribed and salient time-frequency peaks (TF peaks) in the spectrogram, which appear as blob-like regions of increased activity in the 2D image (Figure 1a). This is not surprising, as transient oscillations in the time domain, by definition, will appear as salient TF peaks in the spectrogram [24, 26]. It is also apparent, however, that there are other TF peaks within the sigma range that seem morphologically similar but not scored as spindles. We henceforth refer to all observed *sigma-range time-frequency peaks* as TFσ peaks.

Using several data sets (see Methods), we confirmed that TFσ peaks are abundant and ubiquitous in PSG recordings (Figure 1b, between dashed lines), typically occurring at rates several times higher than the reported 2.5–3 events/minute rate of traditional spindles, regardless of referencing scheme. Furthermore, TFσ peaks not scored as spindles appeared to have strong morphological similarity to those occurring during spindle times, but with a wider range of power.

These observations beg the question: Are classically identified spindles a subset of a broader class of EEG events? We systematically address this question in the following sections.

### Hand-scored spindles form a subset of hand-scored TFσ peaks

#### Hand-scored spindles coincide with hand-scored TFσ peaks

In order to characterize the relationship between spindles and TFσ peaks, we first compared the gold standard time domain hand-scored spindles against hand-scored TFσ peaks. We examined six 30-minute recordings of central channel (C3) EEG during NREM from the DREAMS Sleep Spindle Database [33], which uses contralateral mastoid referencing. To do so, we visually scored TFσ peaks on sleep EEG multitaper spectrograms using a custom program created in MATLAB [40]. Figure 1a illustrates the hand-scored TFσ peaks (dashed boxes) identified on a spectrogram in relation to spindles during one 30-second epoch. We then compared the TFσ peaks with hand-scored spindle times provided by the database, computing event rates and confusion matrix statistics for each record, which are summarized in Table 1.

Overall, the results in Table 1 confirm our initial observations regarding rates, showing that for the same recordings there are often three times as many hand-scored TFσ peaks than hand-scored spindles. The reasonably high precision values (mean 0.85) and temporal overlap percentages (73% average) suggest that most hand-scored spindles coincide with TFσ peaks. Therefore, TFσ peaks capture the majority of the same neurophysiological activity scored as spindles. The greater prevalence of TFσ peaks, unsurprisingly, results in low recall. The F1-scores take the harmonic mean between precision and recall, and therefore they are expectedly moderate for these events.

When we break down all unique hand-scored events, 31.6% (353/1116) are identified as both spindles and TFσ peaks. While 63.9% (713/1116) events are detected exclusively as TFσ peaks, only 4.5% (50/1116) events are identified only as spindles. These fractions agree with the confusion matrix statistics, and they suggest that most spindles can be visualized as distinguishable TFσ peaks and hence manually identified in the time-frequency domain.

#### Hand-scored spindles and TFσ peaks have similar morphology

While the precision scores suggest that the vast majority of hand-scored spindles coincide with hand-scored TFσ peaks, it remains to be shown that the two classes of events reflect similar EEG activity. We therefore compared morphological features of both types of events. To do so, we first visualized the average event spectrograms in Figure 2a (top row of the panel) for the different types of events aggregated across subjects. Mutually detected events exhibit strong transient activity in the spindle frequency range. The events detected only as TFσ peaks are also well-circumscribed TF peaks, but of lesser magnitude and with some bleeding into lower frequencies. In contrast, the events only detected as spindles have diffuse activity in lower frequencies, likely reflecting noisy segments or mis-scoring on time traces. This effect is even more pronounced at the individual subject level (Supplementary Figure S1).

To quantify these observations, we developed a generic procedure to extract features from the most prominent time-frequency local maximum falling within a given hand-scored time segment (see Methods). We characterized the morphology of an event using four properties: prominence (peak power relative to local spectral baseline), duration, central frequency, and bandwidth for each event. As prominence was found to be log-normal distributed, all prominence values are reported in the logarithmic scale.

We compared the feature distributions of the hand-scored spindles and TFσ peaks, which are shown in Figure 2a (bottom panel). There is a marked similarity between the morphological properties of event duration, central frequency, and bandwidth. No significant difference in median was found for central frequency or bandwidth. While median duration differed significantly (paired *t*-test, *p* < 0.05), the effect size was negligibly small at 0.05 second, which is below the temporal resolution of the method. The prominence distributions, on the other hand, varied in structure, with medians differing significantly (paired *t*-test, *p* < 0.001) and an effect size of 2.4 dB higher for spindles than TFσ peaks. Upon further inspection, however, the counts were nearly identical for the most prominent events, diverging in count as prominence decreases, consistent with a biased subsampling rather than a shift. Additional bin-wise tests on the z-scored properties (Supplementary Figure S3a) verified that TFσ peaks significantly outnumber spindles primarily for lower prominence events, while more TFσ peaks are identified across the range for central frequency, duration, and bandwidth.

Together, the above analyses strongly suggest that hand-scored spindles are a morphologically similar subset of hand-scored TFσ peaks in all observed aspects except for prominence. Critically, even though the prominence of hand-scored spindles tends to be higher, the associated prominence distribution is completely nested within the shape of distribution of TFσ peaks, converging with increasing prominence, suggesting a potential sampling bias rather than an intrinsic difference. Thus, we found no evidence to suggest that hand-scored spindles constitute a distinct cluster of events from TFσ peaks.

### Auto-detected spindles show stronger agreement with hand-scored TFσ peaks

To enhance the generalizability of the above results, which may be limited due to the short 30-minute recordings in the DREAMS database, and to reduce the subjectivity due to human scoring, we performed the same analyses in a separate data set, using automated scoring of spindles. For automated scoring, we chose the well-established detector from Wamsley et al. [34], which was rigorously shown to be the most faithful surrogate for human hand-scoring in a large-scale comparison study of numerous methods [17]. We analyzed full-night central channel (C3) EEG recordings from 6 subjects randomly sampled from the original Wamsley study [34], which uses linked-mastoid referencing. For each record, we performed automated spindle detection as well as hand-scored TFσ peaks during all N2 epochs, expanding the number of events analyzed by more than 10-fold (~15.5k events) over the previous analyses. We then repeated the confusion matrix and morphological analyses on this independent and differently referenced dataset.

The results replicate nearly every pattern observed with hand-scored spindles. The event rates in Table 2 again show that hand-scored TFσ peaks are, on average, approximately three times more prevalent than auto-detected spindles (mean rate: 10.4 vs. 3.4 events/minute). Confusion matrix statistics shown in Table 3, 3A recapitulate those for hand-scored spindles, now with an almost perfect precision in some subjects (mean: 0.96), strengthening the claim that traditional spindles are indeed manifested as TFσ peaks in the time-frequency domain. Morphological analyses confirm the results of hand-scored spindles, with the increased sample size clarifying the event similarities (Figure 2b, top panel) and enhancing the clear distributional nesting of features (Figure 2b, bottom panel). As before, there is a significant difference (paired *t*-test *p* < 0.001) and large effect size (3.1 dB) in the medians of prominence, with a markedly skewed distribution for auto-detected spindles matching TFσ peaks almost identically at higher values. Medians of duration, central frequency, and bandwidth showed significant but negligible effect sizes of 0.02 second (paired *t*-test *p* < 0.01), 0.2 Hz (paired *t*-test *p* < 0.01), and 0.01 Hz (paired *t*-test *p* < 0.05), respectively—all well below the temporal and frequency resolutions of the feature extraction method. Bin-wise tests on z-scored properties replicated the same pattern previously observed for hand-scored spindles (Supplementary Figure S3b).

Overall, these results confirm that both hand-scored and auto-detected spindles are subsets of TFσ peaks, with consistent patterns observed across independent datasets and with different referencing schemes. This continues to show that there is no evidence to suggest that spindles constitute a distinct class of events from TFσ peaks.

### Auto-detected spindles converge towards TFσ peaks with relaxed rarity assumption

If there is nothing intrinsic in the data to distinguish spindles from TFσ peaks, then it follows that standard automated methods should identify many of the same events. Why then, do the spindle rates of automated detectors vary so much from TFσ peaks? The majority of automated detectors work by selecting time segments in which some metric of EEG magnitude (e.g. amplitude, spectral power, spectral coefficients, etc.) within a selected frequency range exceeds a fixed threshold. Crucially, the value chosen for the threshold places an implicit rarity assumption on the events detected. For example, placing a threshold at the 95th percentile assumes that only 5% of the observed data are valid detectable events. When this threshold is optimized for concordance with human scorers, this value may be set artificially high to produce rates similar to those observed in hand-scoring.

**Figure 3.**
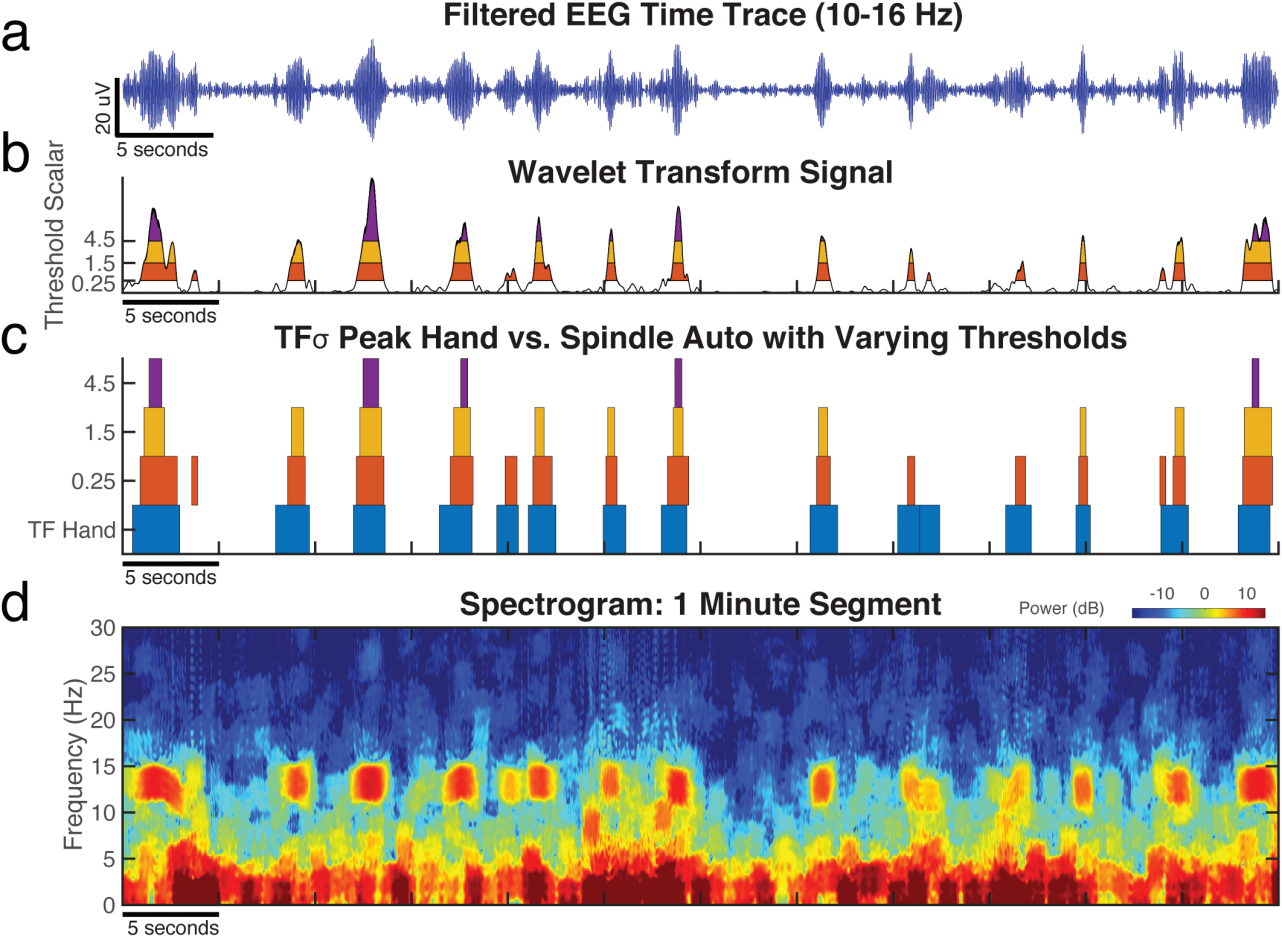
Events identified by the spindle auto-detector (“Sp Auto”) converge towards hand-scored TFσ peaks (“TF Hand”) when the rarity assumption (auto-detector threshold) is relaxed. For an example 60-second segment of C3 data during N2 sleep, we show (a) the EEG time trace filtered to 10–16 Hz, (b) the wavelet magnitude statistic (black curve) computed by the auto-detector, and (d) the EEG spectrogram. When the detection threshold was lowered, more events with lower wavelet magnitude were detected. Colored bands in (b) and (c) indicate time intervals over which individual spindles would be detected by the detector with thresholds set to 0.25 (red), 1.5 (orange) and 4.5 (purple) times the mean amplitude. (c) As the threshold is decreased, the time intervals selected by the spindle auto-detector converge towards the intervals of hand-scored TFσ peaks (blue). (d) Salient TFσ peaks can be easily observed in the spectrogram, which were hand-scored in the time-frequency domain and matched by auto-detected spindles with a threshold of 0.25 as shown in (b) and (c).

To explore the degree to which the imposed rarity assumption is driving the observed difference between spindles and TFσ peaks, we examined the effect of stepping down the amplitude threshold of the automated detector. Figure 3 demonstrates that as the threshold is lowered, more events are detected. For this segment, when the threshold scalar is reduced to 0.25, all of the hand-scored TFσ peaks are also identified by the automated detector. This suggests that TFσ peaks could potentially be detected using traditional methods through a relaxation of the rarity assumption.

To explicitly test this hypothesis, we adjusted the detector threshold for each subject to maximize the F1 score, which balances precision and recall, against the hand-scored TFσ peaks. This approach directly mirrors the analyses in Warby et al., in which the thresholds were optimized with respect to expert hand-scored spindles. The event rates in Table 2 and confusion matrix statistics in Table 3, 3B verify that threshold reduction on the automated detector can well approximate hand-scoring of TFσ peaks. Interestingly, the F1 scores were high (mean: 0.84), exceeding the optimized F1 value (0.68) reported when comparing the same detector to hand-scored spindles [17]. Similar results were achieved using a single group-optimized threshold across all subjects (Supplementary Table 4). These results suggest not only that the automated detector converges towards TFσ peaks with a relaxed rarity assumption, but also that it is better at finding TFσ peaks than hand-scored spindles.

Thus, we show that spindles differ from TFσ peaks during detection only by an arbitrary rarity assumption inherited from the history of hand-scoring spindles. There is a seamless continuum between spindles and TFσ peaks on the wavelet signal used for automated detection, which is consistent with the completely nested distributions of prominence observed before (Figure 2, a and b).

### Unsupervised clustering reliably detects TFσ peaks

**Figure 4.**
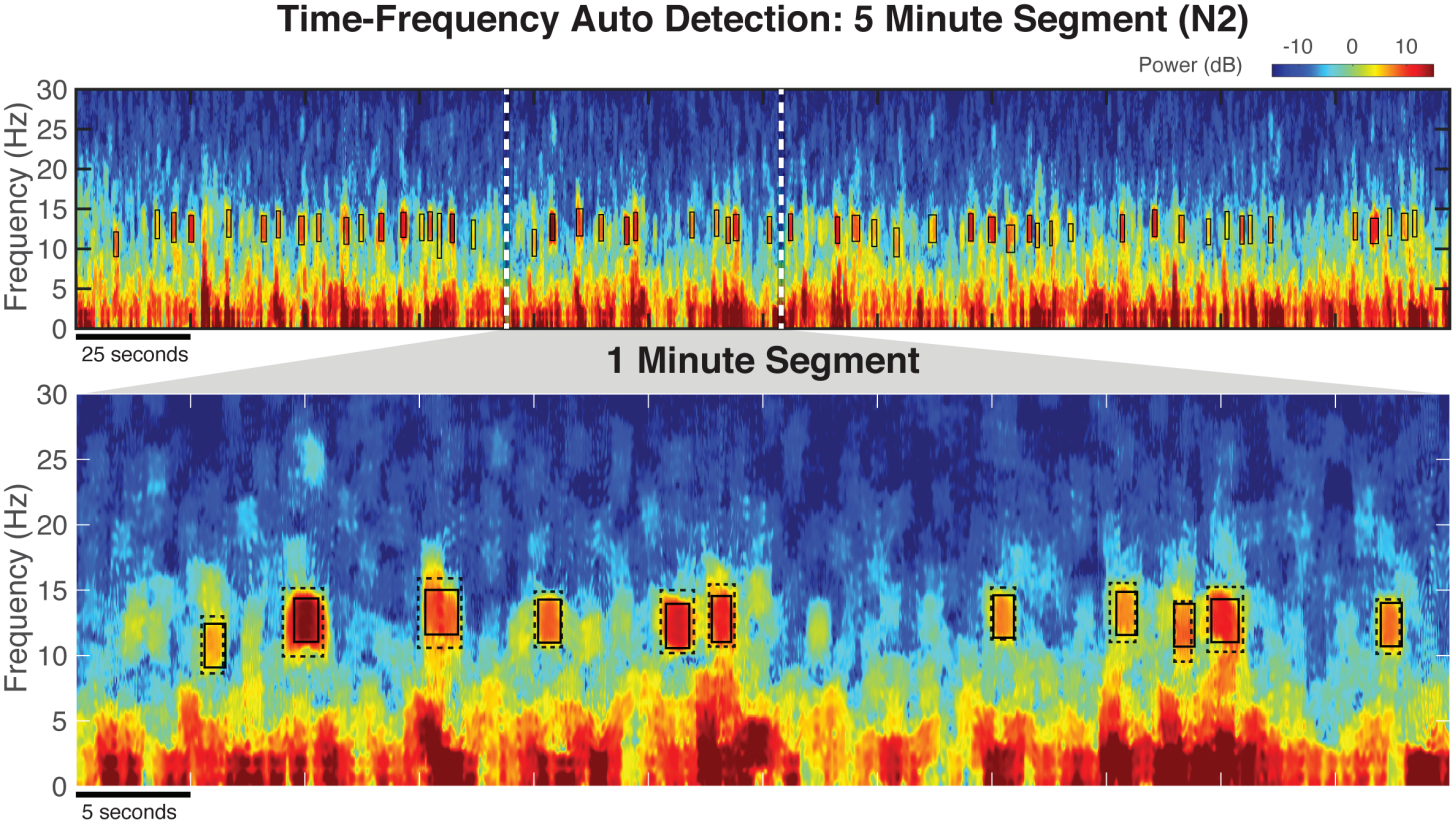
Automated detection of TFσ peaks using unsupervised clustering achieves comparable performance as human hand-scoring. (Top) Auto-detected TFσ peaks (“TF Auto”) marked in solid black boxes reliably extract salient TFσ peaks as can be seen in the spectrogram on a 5-minute segment of C3 data of N2 sleep. (Bottom) Zoomed-in view with hand-scoring of TFσ peaks (“TF Hand”) overlaid in dotted gray boxes demonstrates the close resemblance of the proposed automated detection algorithm to hand-scoring. This example, along with the morphological similarities shown in Figure 2c, illustrates the feasibility of reliably auto-detecting TFσ peaks without the need of an a priori defined fixed threshold.

Just as hand-scoring introduces human subjectivity for spindles, the ability to automatically detect TFσ peaks is necessary for applications to larger datasets and for improved analyses of generalizability. To address this need, we developed an unsupervised clustering approach to automatically detect TFσ peaks. To do so, we performed two-class k-means clustering on the prominence values from all time-frequency local maxima in the sigma range as extracted using our generalized method from the morphology analyses (see Methods). This method determines the difference between noise maxima in the spectrogram and salient TFσ peaks using the data alone, without the need of hand-scored events for training. Figure 4 illustrates that the proposed algorithm can reliably detect TFσ peaks with objective localization of 2D local maxima bounds.

We verified the performance of this automated algorithm in the 6 subjects with available hand-scored TFσ peaks. Comparable event rates to TFσ peak hand-scoring were obtained (Table 2) but with a reduced variability in the automated method at SDs of 0.6 vs. 1.8. Confusion matrix statistics confirm a good performance of the algorithm (Table 3c), with high precision (mean: 0.83) and recall (mean: 0.93) values. The average spectrograms (Figure 2c) show the expected morphology of TFσ peak activity. Analyses of event properties shown in Figure 2c found no major pattern of difference, verifying the equivalence of the automated algorithm to hand-scoring TFσ peaks. Most notably, without any use of scored events, every subject’s F1 score was higher than those derived from the optimized spindle detector. That is, the unsupervised TFσ peak detector produced better matches to hand-scored TFσ peaks than the best possible spindle detector threshold, using the structure of the data alone to separate signal from noise.

### Automated detection of both spindles and TFσ peaks in a larger dataset confirms findings

Equipped with automated detection methods in both time and time-frequency domains, we analyzed recordings from two consecutive nights in 17 subjects (34 total nights). We present the results from the second night. Event rates were comparable with the previous analyses, with mean rates of 9.8 and 3.1 events/minute for TFσ peaks and spindles, respectively. A breakdown of event features for the two methods and average spectrograms repeats the same patterns observed before in this fully automated dataset (Figure 2d) Now with greatly increased numbers of events (~39.0k events), the morphology analysis shows the nesting of features even more clearly. Spindles form a subset of TFσ peaks with high prominence values (paired *t*-test *p* < 0.001, 3.3 dB effect size), while being similar to TFσ peaks on the other three properties with statistically significant but small effect sizes (duration medians differ by 0.02 second; central frequency medians differ by 0.3 Hz; bandwidth medians differ by 0.03 Hz, all *p* < 0.001). Similar results were obtained from all analyses applied to data from the first night (Supplementary Figures S5 and S6).

For central frequency, we observed that auto-detected TFσ peaks have a second peak just above 10 Hz, which likely corresponds to lower-frequency “slow” spindles [39, 41]. While the discussion of the distinction between fast and slow spindles is beyond the scope of this study, these results highlight that the Wamsley detector has a preference for events closer to the center of the frequency range, due to the attenuation of power at the frequency boundaries of 10–16 Hz, which were the same for both methods (see Methods). Regardless, lower-frequency events were not observed in all subjects, comprised a minority of detected events, and did not show up as a secondary peak on z-scored distributions (Supplementary Figure S3d). In addition, bin-wise tests provide strong evidence that these events are not the driving source of the rate differences observed (Supplementary Figure S3d), but rather reveal another facet of sampling bias inherent in standard methods.

Taken together, fully automated TFσ peak and spindle detection in this larger dataset further confirmed our previous findings. In fact, we observe that the distributional properties from hand-scored TFσ peaks and spindles are replicated and enhanced in the auto-detected TFσ peaks and spindles, which come from different subjects, datasets, and were recorded under different referencing schemes—thereby verifying the robustness of TFσ peaks.

### TFσ peak and spindle rates are correlated across subjects

**Figure 5.**
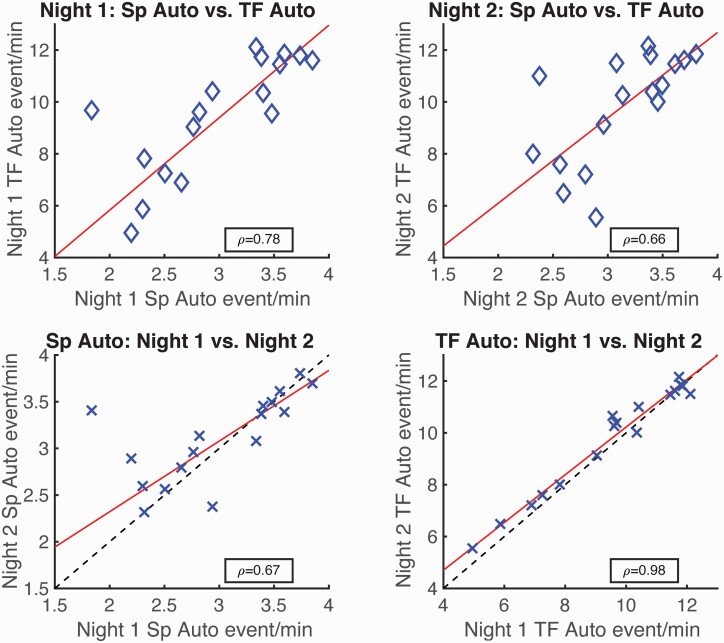
Correlations of auto-detected TFσ peak (“TF Auto”) and spindle (“Sp Auto”) event rates show greater night-to-night stability in TFσ peaks. Using data from two nights in 17 subjects, (top row) significant correlations were found between Night 1 spindles vs. Night 1 TFσ peaks (left) and Night 2 spindles vs. Night 2 TFσ peaks (right). (Bottom row) Significant correlations were also found for intra-individual rates comparing Nights 1 and 2 for spindles (left) and TFσ peaks (right). Correlation was significantly higher (permutation test, *p* < 0.05) for TFσ peaks compared to spindles. Red lines indicate linear regressions using robust fitting procedures (bisquared). Dashed lines (bottom row) indicate perfect correlation (*y* = *x* line). These results suggest that TFσ peaks provide a more reliable characterization of an underlying process that is highly stable within an individual across the two nights.

To explore the relationship of spindles and TFσ peaks within subjects, we performed a cross-subject correlation analysis, which is shown in Figure 5 (top row). Moderate-to-strong correlation coefficients were obtained for both nights (night 1: ρ=0.78, *p* < 0.01, night 2: ρ=0.66, *p* < 0.01), suggesting the underlying processes captured by the two types of events covary within individuals. We also conducted linear regressions across subjects and nights in order to quantify the relationship between the rates produced by both methods. For both nights, slope parameters were ~3 (night 1: 3.6 ± 1.1, night 2: 3.3 ± 1.8) with non-significant intercepts, confirming the ratio of TFσ peaks over spindle counts observed before. Taken together, these patterns provide evidence for a common neurophysiological process underlying TFσ peaks and spindles.

### TFσ peaks exhibit stronger night-to-night stability than spindles

Given that TFσ peaks appear to be a verifiable superset of spindles, do they provide a more robust characterization of subject-specific activity? Spindle rates are known to be a trait-like characteristic [39], which suggests that spindle rates should be similar for separate nights from the same subject. In Figure 5 (bottom row), we show the cross-night correlations of spindles as well as TFσ peaks for the 17 subjects. While the night-to-night rates of spindles have medium-high correlation (ρ=0.67, *p* < 0.01), the rates of TFσ peaks have near-perfect agreement across the nights (ρ=0.98, *p* < .001), showing a significant improvement (permutation test: *p* < 0.05) over spindles. Linear regressions reveal the same pattern when comparing night-to-night rates across methods. While the slope for sleep spindles is 0.76 ± 0.22 (*r*^2^ = 0.72), for TFσ peaks the slope improves to 0.92 ± 0.09 (*r*^*2*^ = 0.96) and is not significantly different from the y = x line. These findings suggest that TFσ peak rates have much stronger intra-individual stability than spindle rates.

## Discussion

Overall, these results provide compelling evidence across multiple data sets and subjects that TFσ peaks constitute a morphologically similar superset of spindles. Moreover, we find no evidence to suggest that TFσ peaks are intrinsically distinct from spindles, which appear to simply be a biased subsampling of TFσ peaks. Thus, we can find, at present, no empirical justification for viewing spindles and TFσ peaks as two separate classes. Moreover, TFσ peaks occur at a greater rate, covary with spindles across subjects, and have improved night-to-night stability. Thus, we conclude that TFσ peaks represent a more robust and comprehensive representation of the observed neurophysiological activity than traditionally defined spindles.

Our results tie together many threads from previous approaches and studies. From a methodological perspective [2, 4], it follows that time domain hand-scoring is necessarily biased towards more easily detected visible events, which has established a precedent for event rarity that was built into automated detectors. Moreover, there do exist automated scorers with more inclusive detection methods [11, 17, 42, 43], which produce rates comparable with those found for TFσ peaks in this study. Additionally, others have sought to explicitly characterize transient oscillatory events more generally [27, 28, 30, 31, 39], expanding the concept of spindles to a larger class. From an empirical perspective, there is direct evidence of spindle events occurring in both humans and rodents at the rate of TFσ peaks. In particular, average neocortical spindle densities of 6.8 (slow spindle) and 10 (fast spindle) events/minute were reported in human intracranial studies [44]. Additionally, there is evidence that optogenetic stimulation of the rodent thalamic reticular nucleus (TRN) can evoke neocortical spindles within ~2.5 seconds of a spontaneous spindle, suggesting no intrinsic mechanistic limitation or refractory period preventing rates of up to ~25 events/minute [45]. Thus, the rates observed for TFσ peaks are physiologically plausible and consistent with activity that could be generated by known neurophysiological processes. Additionally, the strong night-to-night consistency in TFσ peak rate extends the trait-like individual differences observed within average power spectra of sleep EEG [46, 47] to discrete events, as well as reflects the demonstrated heritability of spindle morphological features and the broader concept that information content can be found in “low-amplitude” spindles [48].

What then could be the mechanistic basis for the “spindle-like” activity driving the production of TFσ peaks? As the analyses herein compare EEG events, it remains to be proven that the neuronal processes underlying TFσ peak activity share the same thalamic origin and the involvement of both the thalamic reticular nucleus and thalamocortical loops as described for spindles [49]. Nevertheless, there are several candidate explanations that could account for the abundant spindle-like events observed through TFσ peaks. The simplest possibility is that TFσ peaks are generated by the exact same mechanism as spindles and that any differences are purely a consequence of selection bias. Spindle power measured at the scalp can vary with the degree with which a spindle is local or global [50, 51]. Alternatively, high-powered spindles emanating from cortical locations more distant from the recording electrode might appear to be weaker when propagated via volume conduction [52, 53]. Mechanistically, a distinction between “core” and “matrix” spindles has been proposed to account for the difference between EEG spindles and MEG spindles [54, 55], which are less prominent but otherwise morphologically identical [56]. Further study is therefore required to clarify the source of the spindle-like activity underlying TFσ peaks.

Likewise, the functional significance of an expanded definition of spindle-like activity over spindles needs active exploration. Our results showing improved intra-individual stability suggest instances in which the superset of TFσ peaks can provide added information and greater statistical power relative to spindles. It will be vital to characterize the relationship between TFσ peaks and other known correlates of spindle activity, such as memory consolidation, aged-related changes in sleep, and thalamocortical networks in diseases such as Alzheimer’s disease and schizophrenia. Additionally, studies of large populations [48] will further assess the generalizability of these results, as well as connections to epidemiological and demographic variability.

In this article, we have provided evidence of an expanded notion of the spindle phenomenon in the time-frequency domain. In doing so, it is abundantly clear that the current gold standard falls short of accurately capturing the EEG activity underlying the spindle phenomenon. The construct of TFσ peaks offers a far more principled starting point for a comprehensive characterization of spindle-like and related neuronal activity within the existing PSG recording paradigm. Future research should consider this broader view and previous findings on sleep spindles should be reexamined within this context. Ultimately, this work constitutes a beginning, rather than an end point for an improved characterization of spindle activity, as there are many more dimensions of observation to explore. More generally, this work serves to highlight the need to reevaluate long-standing notions in the sleep field in the light of new approaches, with a goal of rooting our understanding in as objective and principled an analysis of the data as possible.

## Supplementary Material

zsab099_suppl_Supplementary_Material

## Data Availability

The data from the DREAMS Sleep Spindle Database are available at http://doi.org/10.5281/zenodo.2650142. The data from Wamsley et al. were provided by Dr. Dara Manoach by permission. *Code Distribution*: All functions employed for multitaper spectrogram estimation, artifact rejection, event property extraction, hand-scoring of TFσ peaks, and automated TFσ peak detection using k-means clustering are available at: http://sleepEEG.org/TFsigma_peaks. For auto-detectors of spindles, see Warby et al. [17].
