# Supplementary material for: Sleep spindles comprise a subset of a broader class of electroencephalogram events
Source: Sleep. 2021 Apr 15;44(9):zsab099. doi: 10.1093/sleep/zsab099 (PMC8436142; doi:10.1093/sleep/zsab099)
Supplement: zsab099_suppl_Supplementary_Material [file zsab099_suppl_supplementary_material.docx]

# Sleep spindles comprise a subset of a broader class of electroencephalogram events

**Supplementary Material**

Tanya Dimitrov^1^* & Mingjian He^1,2,3^*, Robert Stickgold^4,5^, Michael J. Prerau^1^**

1. Brigham and Women’s Hospital, Division of Sleep and Circadian Disorders, Boston, MA, USA
2. Harvard-MIT Health Sciences and Technology, MIT, Cambridge, MA, USA
3. Department of Anesthesia, Critical Care and Pain Medicine, Massachusetts General Hospital, Harvard Medical School, Boston, MA, USA
4. Beth Israel Deaconess Medical Center, Department of Psychiatry, Boston, MA, USA
5. Harvard Medical School, Department of Psychiatry, Boston, MA, USA

*These authors contributed equally to the work.

**Corresponding Author:

Michael J. Prerau

[mprerau@bwh.harvard.edu](mailto:mprerau@bwh.harvard.edu)

# Supplementary Material

**Time-frequency event property extraction algorithm**

The “frequency step” is based on the concept of peak prominence, which stems from geological studies [1] and measures of the height of a peak relative to the lowest contour line around it that excludes higher peaks. In essence, peak prominence computes the magnitude (height, power, amplitude) of a peak relative to a hyper-local baseline. Thus, prominence provides a better description of  EEG spectral peak magnitude than absolute power or a global baseline, accounting for instantaneous offsets in power as well as for the background 1/f slope in the frequency dimension [2]. We first estimate the multitaper spectrogram using the same parameters described above and apply the MATLAB *findpeaks()* function for each spectrum, estimating prominence, frequency at maxima, and bandwidth for all peaks in the frequency range 6-30 Hz. This broad range was to provide the data required to accurately compute prominence while excluding confounding effects from slow/DC activity. We then select peaks within 9-17 Hz with maximal prominence, which we term the *max-prominence peak* for each time window. This frequency range is 1 Hz wider than the intended range (10-16 Hz) in order to conservatively account for main lobe bandwidth. We then construct a *prominence time series* by concatenating prominence values of all max-prominence peaks across time windows. Time windows containing artifacts, falling outside of N2 sleep, or having no valid peak in the 9-17 Hz range were marked as missing values. Therefore, the frequency prominence time series spanned the full night, sampled at intervals of the step size (0.05 s) used in multitaper spectrogram estimation.

For the “time step”, we perform peak extraction again on the prominence time series. We smooth the frequency prominence with a 0.3 s moving average window to avoid small inflection points being considered as peaks in the next step. We then apply the *findpeaks()* function on the smoothed time series to identify prominent peaks along the time dimension. As this second round of peak finding operates on a time series of prominence values, the computed “prominence of prominence” provided a single numerical value quantifying the distinctiveness of a time-frequency peak (local maximum). We converted the obtained (time) prominence values to the logarithmic scale due to observed log-normal distributions.

Having identified salient time-frequency peaks in spindle frequency range across the night, we use the parameters of these peaks to extract event properties. For an event detected by any method (by hand-scoring or automated detectors, in the time or time-frequency domain), we defined the scored interval using the marked onset and offset times of the event. The time-frequency peak with temporal maximum closest to the midpoint of the scored interval was assigned to the event. For a given peak in the prominence time series, with the maximum at time *t*, we extract the following four properties of the assigned event:

*Prominence = natural log of the peak prominence*

*Duration = half-prominence width of the peak*

*Central Frequency = frequency of the max-prominence peak of the spectrum at t*

*Bandwidth = half-prominence width of the max-prominence peak of the spectrum at t*

If multiple peaks occurred during the event interval, the local maximum with the highest prominence was assigned. If no local maximum in the prominence time series overlapped with the scored interval (<2% for all methods and subjects), features were determined using the closest peak available (always occurred within 5 s from the event interval midpoint) based on the following rules. For an event with the event interval midpoint, *t_m_*, and a corresponding peak closest to the event with its maximum at time *t* in the prominence time series:

*Prominence = natural log of the peak prominence – (prominence at t – prominence at t_m_)*

*Duration = event offset time – event onset time*

*Central Frequency = frequency of the max-prominence peak of the spectrum at t_m_*

*Bandwidth = half-prominence width of the max-prominence peak of the spectrum at t_m_*

Using the detected properties, we can restrict detected events to match the specific assumptions inherent in specific detectors against which the results are being compared. In this case, we remove all events with duration less than 0.3 s and a central frequency outside of 10-16 Hz in order to match with the methods used in Wamsley et. al.

In summary, this algorithm identifies the most prominent, well-defined time-frequency peak, within a given time period and extracts the four associated properties. Since the only needed inputs are event onset and offset times, this algorithm is agnostic to the details of how an event is detected. Thus, properties can be extracted equitably for all methods investigated in the present study, allowing fair comparisons across time and time-frequency domains. It should be noted this approach does not explicitly handle multiple overlapping time-frequency events, however simultaneously co-occurring events are rare in the sigma range, thus making this an appropriate method in this application.

**DREAMS subjects’ individual average spectrograms**

TFσ peaks were hand-scored in the 6 subjects from the DREAMS Sleep Spindle Database [3] and compared with hand-scored sleep spindles provided by the database. As can be seen in Figure S1, very few events are scored only as spindles. Those that were scored only as spindles (Sp Hand Only) tend to have diffuse activity in lower frequencies. While the cross-subject average (Figure 2a, top row) shows some vertical smearing for the hand-scored TFσ peaks (TF Hand Only), we can now see that it is mostly due to the contributions of subjects 2 and 4, for whom lower frequency TFσ peaks were scored. A detailed breakdown of the numbers of events are shown in Supplementary Table 1.

**Supplementary Table 1.** **Numbers of detected events in 6 subjects from DREAMS Sleep Spindle Database for hand-scored TFσ peaks and spindles**

| **Subject ID** | **Total** | **Both** | **TF Hand Only** | **Sp Hand Only** | **TF Hand All** | **Sp Hand All** |
| --- | --- | --- | --- | --- | --- | --- |
| **1** | 250 | 102 | 136 | 12 | 238 | 114 |
| **2** | 179 | 43 | 128 | 8 | 174 | 51 |
| **3** | 126 | 38 | 83 | 5 | 122 | 43 |
| **4** | 180 | 15 | 156 | 9 | 172 | 24 |
| **5** | 205 | 76 | 120 | 9 | 197 | 85 |
| **6** | 176 | 79 | 90 | 7 | 172 | 86 |

This table shows the breakdown of events detected by hand-scoring of sleep spindles (SP Hand) and TFσ peaks (TF Hand) in the DREAMS database. Total event counts refer to the total number of events detected regardless of method, which is composed of events labelled as both TFσ peaks and spindles (Both), events only labelled as TFσ peaks (TF Hand Only), and events only labelled as spindles (Sp Hand Only). As the low numbers of events only scored as spindles suggest, TFσ peak hand-scoring can identify most of the hand-scored spindles. Confusion matrix statistics are shown in Table 1 of main manuscript.

**
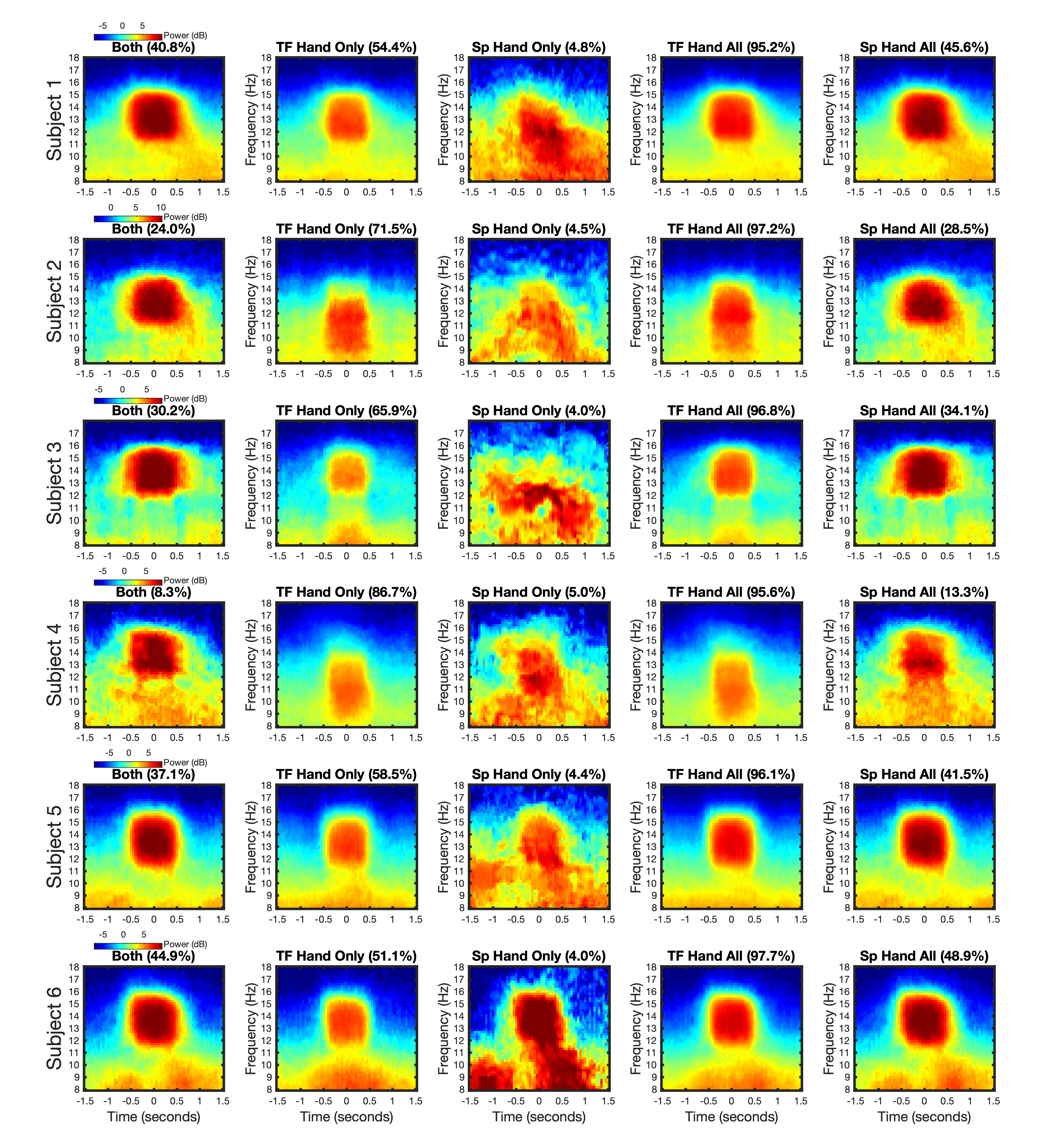
**

Figure S1: Hand-scored TFσ peaks and hand-scored spindles show morphological similarities. Median event spectrograms are shown for hand-scored TFσ peaks (“TF Hand”) and hand-scored spindles (“Sp Hand”) for all 6 DREAMS subjects. Events are grouped by: events detected by both methods (“Both”), events detected by a single method (“Only”), and all the events detected by a given method (“All”). The percentages of events out of total number of unique events are reported.

**Control subjects’ individual average spectrograms**

The 6 control subjects with full night hand-scoring of TFσ peaks were compared with auto-detected spindles. Figure S2 shows patterns similar to those previously observed in Figure S1, but with a clearer delineation of the weaker but well-circumscribed events detected only as TFσ peaks. Supplementary Table 2 shows the breakdown of event numbers.

**Supplementary Table 2.** **Numbers of detected events in full night recordings from 6 subjects for hand-scored TFσ peaks and auto-detected spindles**

| **Subject ID** | **Total** | **Both** | **TF Hand Only** | **Sp Auto Only** | **TF Hand All** | **Sp Auto All** |
| --- | --- | --- | --- | --- | --- | --- |
| **1** | 2540 | 896 | 1496 | 148 | 2392 | 1027 |
| **2** | 2616 | 730 | 1882 | 4 | 2612 | 735 |
| **3** | 3267 | 993 | 2269 | 5 | 3262 | 986 |
| **4** | 2179 | 705 | 1459 | 15 | 2164 | 691 |
| **5** | 2084 | 757 | 1296 | 31 | 2053 | 784 |
| **6** | 2816 | 840 | 1954 | 22 | 2794 | 834 |

This table shows the breakdown of events detected by hand-scoring of TFσ peaks (TF Hand) and automated detection of spindles (Sp Auto). These results are obtained in 6 subjects from the control subject cohort with full-night recordings and hand-scored TFσ peaks and different from the 6 segments in DREAMS database shown in Supplementary Table 1. Total event counts refer to the total number of events detected regardless of method, which is composed of events labelled as both TFσ peaks and spindles (Both), events only labelled as TFσ peaks (TF Hand Only), and events only detected as spindles (Sp Auto Only). The majority of spindles are also scored as TFσ peaks by hand-scoring, which is consistent with the pattern observed in Supplementary Table 1, except now with much more events. Confusion matrix statistics for this comparison are shown in Table 3A of main manuscript.

**
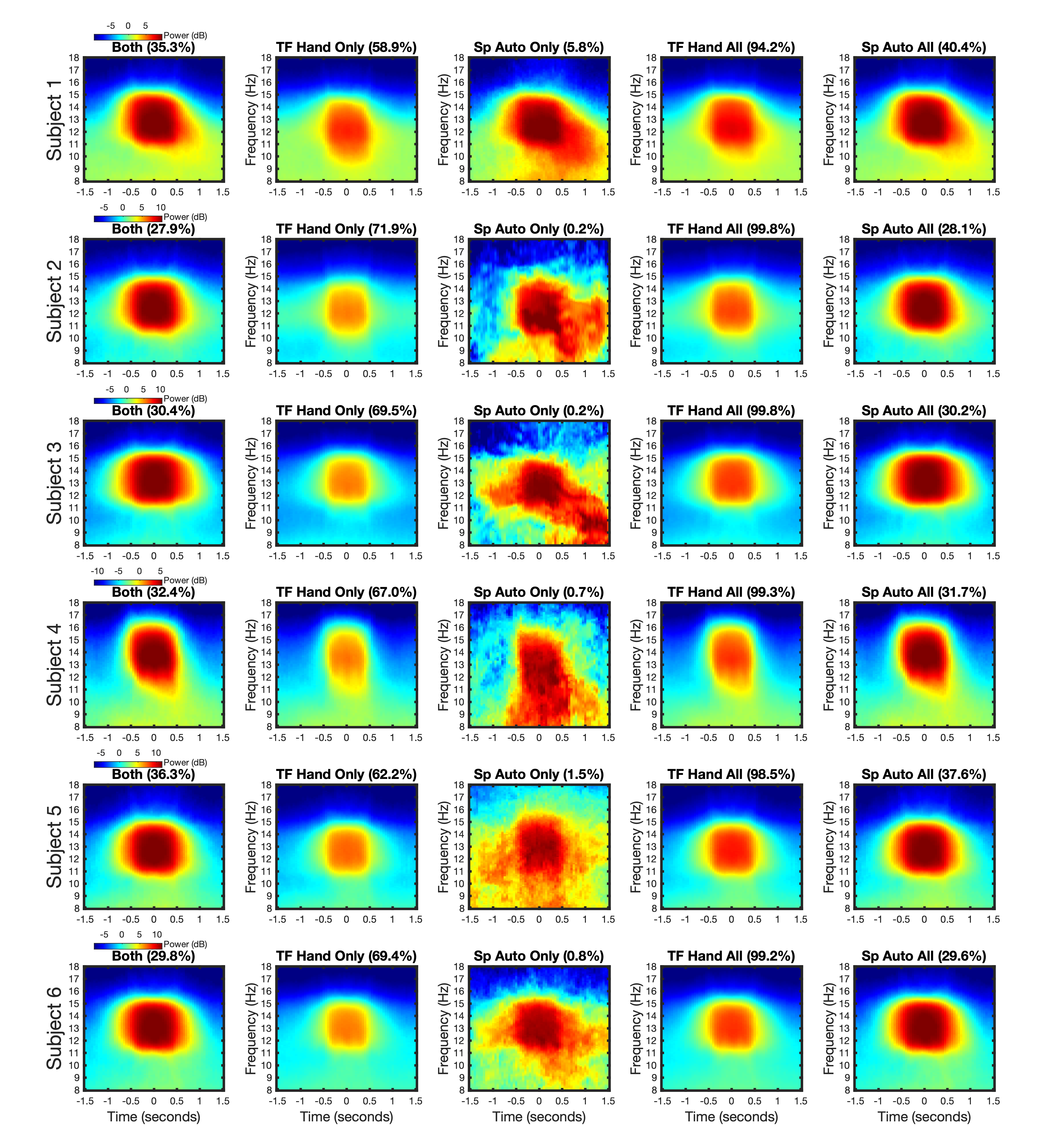
**

Figure S2: Hand-scored TFσ peaks and auto-detected spindles show morphological similarities. Median event spectrograms are shown for hand-scored TFσ peaks (“TF Hand”) and auto-detected spindles (“Sp Auto”) for all individual DREAMS subjects. Events are grouped by: events detected by both methods (“Both”), events detected by a single method (“Only”), and all the events detected by a given method (“All”). The percentage of all uniquely events as well as the total number of unique events are reported.

Analysis of Z-scored event properties

As described in the *Methods* section, we conducted binned tests on the z-scored property values to see if the increased number of TFσ peaks with lower prominence was driven by a specific type of events (e.g., slow spindles)**.** Figure S3 shows z-scored property distributions analogous to histograms shown in Figure 2, with corresponding binned tests and boxplots on the z-scored properties.

The top two panels (Figure S3a, S3b) show largely identical results: while TFσ peaks outnumber spindles exclusively in lower prominence events, significantly more TFσ peaks are detected than spindles across the range of the other three properties (duration, central frequency, and bandwidth). This suggests that there isn’t clear separation between TFσ peaks and spindles other than on the prominence dimension. Nevertheless, as the z-scored distributions and Figure 2ab show clearly, the prominence distribution of spindles is completely nested within that of TFσ peaks, consistent with a pattern generated by biased sampling rather than two distinct types of EEG events.

Figure S3c shows the comparability of hand-scored TFσ peaks and auto-detected TFσ peaks, as no consistent difference could be observed for any of the properties except a few sporadic significant bins. This is consistent with the largely overlapping distributions shown in Figure 2c.

Figure S3d shows replicates of the patterns in Figure S3ab with full night recordings from 17 subjects with auto-detected TFσ peaks and spindles. Now with increased sample sizes, the Wilcoxon signed-rank tests reached significance in all bins of most of the properties. However, as can be observed from the boxplots as well as the z-scored histogram distributions, TFσ peaks outnumber spindles primarily in the lower prominence range while having similarly localized but dominating (having greater number of events) distributions compared to spindles in the other three properties. It’s worth noting that the secondary peak at 10 Hz observed in Figure 2d is no longer evident in the z-scored property distribution. This suggests that while there is a cluster of events centered at lower frequencies (presumably slow spindles), these events have varying deviations from subject-specific central frequency distributions of auto-detected spindles, therefore not producing an evident cluster of events in the aggregate z-scored distribution.

Taken together, binned tests complement the median paired-t tests reported in the main manuscript and z-scored histograms complement the aggregate histograms on physical units shown in Figure 2. These results show that the difference between TFσ peaks and spindles was mostly driven by a difference in the prominence value of detected events rather than an inherent separation in event duration, central frequency, or bandwidth. This pattern strongly supports the hypothesis that spindles is a particular subset of TFσ peaks, formed by a biased sampling that selects the most prominent events inherited from the history of hand-scoring spindles and the implicitly imposed rarity assumption of spindle rates.

**
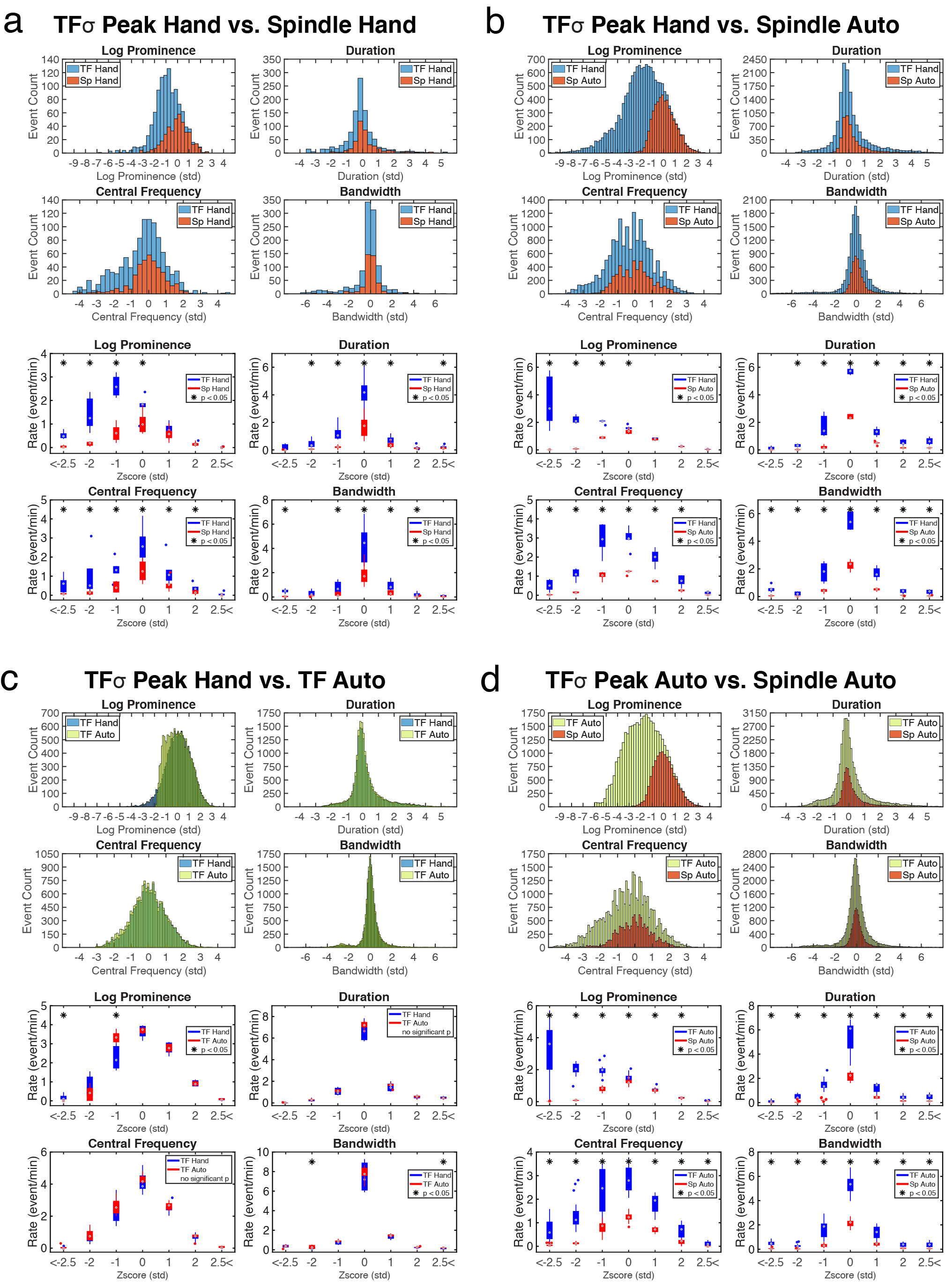
**

Figure S3: Binned tests on z-scored event properties show that TFσ peaks outnumber spindles across a wide range of event morphologies. Binned tests were performed for hand-scored and auto-detected TFσ peaks (“TF Hand/Auto”) and for hand-scored and auto-detected spindles (“Sp Hand/Auto”). Distributions of z-scored event properties (top subpanels), boxplots and non-parametric test significant bins (bottom subpanels) are shown for (a) TF Hand and Sp Hand, (b) TF Hand and Sp Auto (c) TF Hand and TF Auto (d) TF Auto and Sp Auto. Results suggest that the increased number of TFσ peaks with lower prominence is driven by events that are otherwise broadly distributed throughout the morphology space.

**Distribution-based comparisons of event properties**

In comparing TFσ peak and spindle properties, it is tempting to use well-established paradigms for the comparison of statistical distributions. In particular, the Kolmogorov-Smirnov (KS) test is a popular choice when testing whether an empirical distribution differs significantly from a parameterized theoretical distribution based on the differences in the cumulative distribution functions (CDF). However, the two-sample test is a global test on the entire domain of empirical distributions, i.e., as soon as the two distributions differ at any part of the CDFs, the test will reject the null hypothesis and suggest that the two distributions are statistically different from each other. Additionally, the test statistic D has a critical threshold that scales with the product of sample sizes:

$$D_{n,m}^{crit}=c(\alpha)\sqrt{\frac{n+m}{n\cdot m}}$$

where$n,m$ are sample sizes of the two distributions, and $c(\alpha)$ is a function dependent on the chosen significance level, which is set to be 1.358 based on a significance level of 0.05 [4]. This means the critical threshold decreases linearly with the product of the sample sizes, and the test becomes very sensitive to small deviations at even moderate sample sizes, as can be observed directly from the formula. Finally, it is not necessary that the distributions of spindle and TFσ peak properties be identical to show that one is the subset of the other. In particular, biased sampling (which we have strongly concluded is happening in this case) would by definition produce events distributions that were highly dissimilar. Regardless, herein we provide the KS statistic values for each distributional comparison, since they are informative about the maximum deviation in cumulative probability, which provides an upper bound on the degree to which the distributions differ the most.

**Supplementary Table 3. D statistics of two-sample KS tests comparing event property distributions**

|  | **Event Properties** | | | |
| --- | --- | --- | --- | --- |
| **Comparisons** | **Prominence** | **Duration** | **Central Frequency** | **Bandwidth** |
| **TF Hand vs. Sp Hand** | 0.31 | 0.14 | 0.18 | 0.12 |
| **TF Hand vs. Sp Auto** | 0.45 | 0.14 | 0.10 | 0.10 |
| **TF Hand vs. TF Auto** | 0.03 | 0.01 | 0.03 | 0.02 |
| **TF Auto vs. Sp Auto** | 0.35 | 0.13 | 0.16 | 0.11 |

This table shows D statistics of KS tests for event properties of the four sets of method comparisons shown in Figure 2 of main manuscript. The same pattern can be seen across the comparisons of TFσ peaks and spindles regardless of the detection modality (TF = TFσ peaks, Sp = spindles, Hand = hand-scored, Auto = auto-detected). While the two classes of events different significantly with up to ~0.3-0.4 cumulative probability on the dimension of prominence, the events are similar on the other three dimensions. This pattern suggests that TFσ peaks do not differ fundamentally from spindles, except for a broader sampling of weaker events as reflected by the deviation on prominence. In contrast, the cumulative distributions of hand-scored and auto-detected TFσ peaks are very close to each other, verifying the robust performance of the algorithm to emulate hand-scoring of TFσ peaks in the spectrograms.

**Single group-optimized threshold for auto-detection of spindles**

To assess the generalizability of a single threshold to perform well across subjects we computed the threshold that maximizes the mean F1-score across the 6 controls, which was found to be 0.40. This threshold was then applied to the 6 subjects and compared against hand-scored TFσ peaks.

We repeated the confusion matrix comparison with hand-scored TFσ peaks, with the results shown in Supplementary Table 4 below. As can be seen from the high average F1 scores, the group-optimized detector is not much worse than individually optimizing the thresholds (0.82 compared to 0.84). This reinforces our observation that an automated detection algorithm designed for hand-scored spindles can in fact achieve much better results (higher than 0.68 achieved for optimized against hand-scored spindles) when used to approximate hand-scoring TFσ peaks. This result provides indirect evidence that TFσ peaks constitute a more consistent class of EEG events to be detected compared to spindles.

A question then is whether future research when detecting spindles should use a much lower threshold, such as 0.5 rather than 4.5, to be more inclusive of the TFσ peak events identified in this study? To address this question, we repeated the event rate correlative analyses presented in Figure 5 on the events here detected using a single fixed threshold at 0.40 for all 17 subjects across the two nights.

As can be seen in the correlative patterns in Figure S4, while the cross-method correlations are only slightly weaker with this group-optimized Wamsley detector, the strong intra-individual stability of events rates of TFσ peak is completely lost compared to the data-driven clustering detection of TFσ peak (Figure 5). This result serves as a control analysis for the strong cross-night correlation of TFσ peaks, which was not merely an artifact of increased event rates. Similarly high event rates are reported here for group-optimized Wamsley detection, yet the correlation is much weaker. In addition, these findings suggest that lowering the thresholds of automated detectors originally designed to detect spindles on time traces might not produce desirable results of detecting the class of TFσ peak events. One reason for the poor performance of the group optimized detector is that as the threshold gets lower, more noise events are also selected. In contrast, a direct detection of salient TFσ peaks from spectrograms with unsupervised clustering can better identify relevant events by operating in both time and frequency dimensions.

**Supplementary Table 4. Using a single threshold of 0.40 for the 6 subjects with hand-scored TF_σ_ peaks**

|  | **TFσ peak Hand** | **Spindle Auto group-optimized** |  |  |  |  |
| --- | --- | --- | --- | --- | --- | --- |
| **Subject** | **Rate (events/min)** | **Rate (events/min)** | **Precision** | **Recall** | **F1** | **Overlap %** |
| 1 | 8.4 | 11.6 | 0.60 | 0.84 | 0.70 | 67 |
| 2 | 10.9 | 10.7 | 0.83 | 0.81 | 0.82 | 61 |
| 3 | 12.2 | 10.0 | 0.96 | 0.79 | 0.87 | 52 |
| 4 | 9.2 | 10.6 | 0.74 | 0.89 | 0.81 | 52 |
| 5 | 8.9 | 10.0 | 0.83 | 0.92 | 0.87 | 61 |
| 6 | 12.7 | 11.5 | 0.89 | 0.82 | 0.85 | 53 |
| **Mean ± Std:** | **10.4 ± 1.8** | **10.7 ± 0.7** | **0.81 ± 0.12** | **0.84 ± 0.05** | **0.82 ± 0.06** | **58 ± 6.5** |

This table shows the event detection comparisons of hand-scored TFσ peaks and auto-detected spindles using a single uniform adjusted threshold. This threshold is group-optimized such that it maximizes the mean F1-score across the subjects. The 6 subjects analyzed are from the control subject cohort with full-night recordings and hand-scored TFσ peaks and different from the 6 segments in DREAMS database shown in Supplementary Table 1. As shown by the comparable rates, high precision and recall scores, the single group-optimized threshold of 0.40 achieves good correspondence with hand-scored TFσ peaks. Unsurprisingly, the F1 scores are lower than the maximal F1-scores reported in Table 3B with individually optimized thresholds. This result suggests that while the optimal thresholds vary across subjects, using a uniform low threshold can achieve reasonable approximation to hand-scored TFσ peaks. However, as shown in the other analyses below, auto-detected spindles using the group-optimized threshold are less robust than TFσ peaks and display much lower cross-night stability.

**
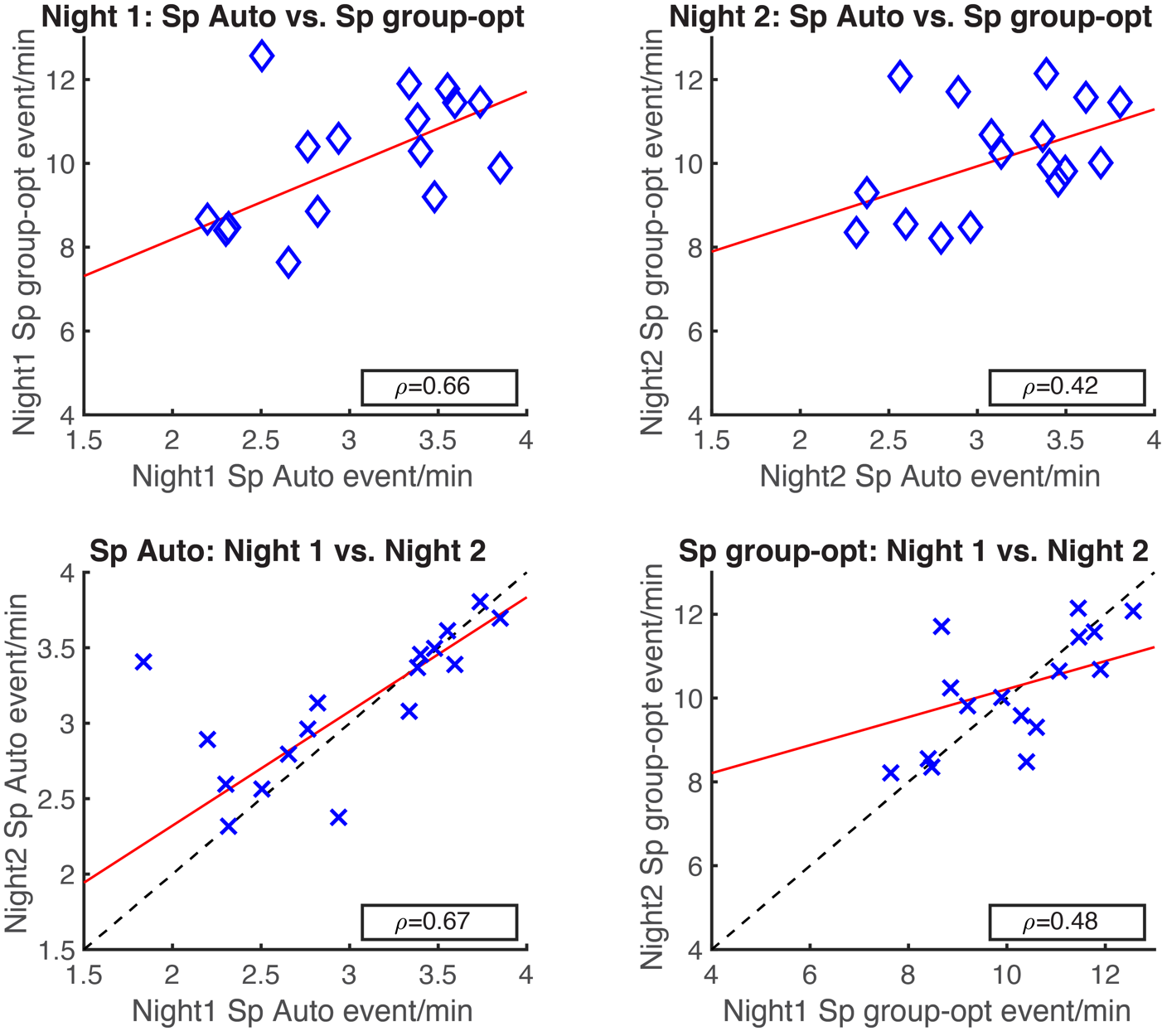
**

Figure S4: Correlations of auto-detected spindle event rates with group-optimized threshold (“Sp group-opt”) no longer show strong night-to-night stability compared to event rates of auto-detected spindles (“Sp Auto”). Using data from two nights in 17 subjects, (top row) significant correlations were found between Night 1 spindles vs. Night 1 group-optimized threshold spindles (left) and Night 2 spindles vs. Night 2 group-optimized threshold spindles (right). (Bottom row) Significant correlations were also found for intra-individual rates comparing Nights 1 and 2 for spindles (left). In contrast to the results found for TFσ peaks, spindles detected with group-optimized threshold (right) no longer exhibit the strong cross-night correlation despite detecting events at similarly high rates as TFσ peaks. Red lines indicate linear regressions using robust fitting procedures (bisquared). Dashed lines (bottom row) indicate perfect correlation (y = x line). These results suggest that lowering the thresholds of automated detectors originally designed to detect spindles on time traces might not produce desirable results of detecting the class of TFσ peak events.

**Control subjects auto-detection results from both nights**

In the main results, we reported the results and analyses on auto-detected TFσ peaks and spindles on the second night to avoid adaptation effects. For completeness, we repeated these analyses on the first night, with highly similar results obtained. This analysis further validates the strong cross-night stability shown in both spindles and TFσ peaks. These results are shown below in Supplementary Tables 5 and 6, as well as Figures S5 and S6.

**Supplementary Table 5. Control subjects Night1: auto-detected TFσ peaks and spindles**

|  | **TFσ peak Auto** | **Spindle Auto** |  |  |  |  |
| --- | --- | --- | --- | --- | --- | --- |
| **Subject** | **Rate (events/min)** | **Rate (events/min)** | **Precision** | **Recall** | **F1** | **Overlap%** |
| 1 | 9.6 | 2.8 | 1.00 | 0.29 | 0.45 | 54 |
| 2 | 11.5 | 3.6 | 0.98 | 0.31 | 0.47 | 51 |
| 3 | 7.3 | 2.5 | 0.82 | 0.28 | 0.42 | 49 |
| 4 | 11.7 | 3.4 | 0.98 | 0.28 | 0.44 | 54 |
| 5 | 5.9 | 2.3 | 0.94 | 0.37 | 0.53 | 55 |
| 6 | 12.1 | 3.3 | 0.99 | 0.27 | 0.43 | 51 |
| 7 | 7.7 | 2.3 | 0.91 | 0.28 | 0.42 | 51 |
| 8 | 11.9 | 3.6 | 1.00 | 0.30 | 0.47 | 52 |
| 9 | 9.0 | 2.8 | 0.88 | 0.27 | 0.42 | 51 |
| 10 | 11.6 | 3.9 | 1.00 | 0.33 | 0.50 | 59 |
| 11 | 5.0 | 2.2 | 0.81 | 0.36 | 0.50 | 55 |
| 12 | 6.9 | 2.7 | 0.99 | 0.38 | 0.55 | 59 |
| 13 | 10.4 | 2.9 | 0.99 | 0.28 | 0.43 | 49 |
| 14 | 9.6 | 3.5 | 0.99 | 0.36 | 0.53 | 54 |
| 15 | 9.7 | 1.8 | 0.59 | 0.11 | 0.19 | 43 |
| 16 | 11.8 | 3.7 | 1.00 | 0.32 | 0.48 | 57 |
| 17 | 10.4 | 3.4 | 0.99 | 0.32 | 0.49 | 56 |
| **Mean ± Std:** | **9.5 ± 2.3** | **3.0 ± 0.6** | **0.93 ± 0.11** | **0.30 ± 0.06** | **0.45 ± 0.08** | **53 ± 4.0** |

This table shows the event detection comparisons of auto-detected TFσ peaks (TFσ peak Auto) and spindles (Spindle Auto) for all 17 subjects during the first night. Confusion matrix statistics treat TFσ peaks as ground truth.

**Supplementary Table 6.** Control subjects Night2: auto-detected TFσ peaks and spindles

|  | **TFσ peak Auto** | **Spindle Auto** |  |  |  |  |
| --- | --- | --- | --- | --- | --- | --- |
| **Subject** | **Rate (events/min)** | **Rate (events/min)** | **Precision** | **Recall** | **F1** | **Overlap%** |
| 1 | 10.3 | 3.1 | 0.99 | 0.30 | 0.46 | 54 |
| 2 | 11.5 | 3.6 | 0.99 | 0.31 | 0.47 | 53 |
| 3 | 7.6 | 2.6 | 0.86 | 0.29 | 0.43 | 49 |
| 4 | 12.2 | 3.4 | 0.99 | 0.27 | 0.43 | 58 |
| 5 | 6.5 | 2.6 | 0.96 | 0.38 | 0.55 | 56 |
| 6 | 11.5 | 3.1 | 0.99 | 0.27 | 0.42 | 54 |
| 7 | 8.0 | 2.3 | 0.95 | 0.27 | 0.43 | 52 |
| 8 | 11.8 | 3.4 | 1.00 | 0.29 | 0.45 | 52 |
| 9 | 9.1 | 3.0 | 0.94 | 0.31 | 0.46 | 55 |
| 10 | 11.6 | 3.7 | 1.00 | 0.32 | 0.48 | 58 |
| 11 | 5.5 | 2.9 | 0.82 | 0.43 | 0.56 | 53 |
| 12 | 7.2 | 2.8 | 0.99 | 0.38 | 0.55 | 60 |
| 13 | 11.0 | 2.4 | 1.00 | 0.22 | 0.36 | 49 |
| 14 | 10.7 | 3.5 | 0.99 | 0.32 | 0.49 | 58 |
| 15 | 10.4 | 3.4 | 0.98 | 0.32 | 0.49 | 54 |
| 16 | 11.9 | 3.8 | 1.00 | 0.32 | 0.48 | 56 |
| 17 | 10.0 | 3.5 | 0.99 | 0.34 | 0.51 | 55 |
| **Mean ± Std:** | **9.8 ± 2.1** | **3.1 ± 0.5** | **0.97 ± 0.05** | **0.31 ± 0.05** | **0.47 ± 0.05** | **54.5 ± 3.1** |

This table shows the event detection comparisons of auto-detected TFσ peaks (TFσ peak Auto) and spindles (Spindle Auto) for all 17 subjects during the second night. Confusion matrix statistics treat TFσ peaks as ground truth.

**
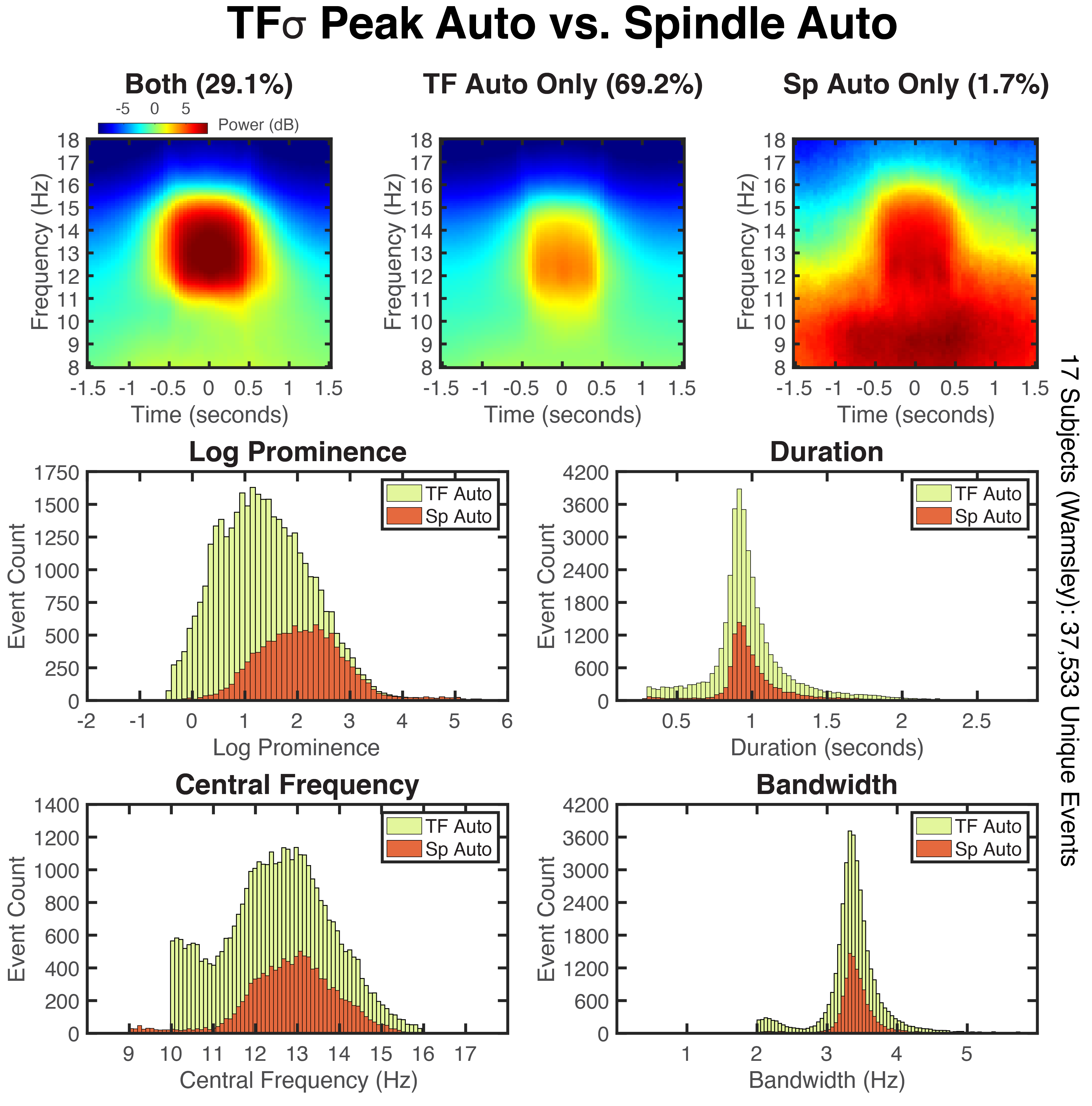
**

Figure S5: Results from the first night replicate the pattern that spindles are a morphologically similar subset of TFσ peaks. We compare the average event spectrograms and morphological property distributions of auto-detected spindles (“Sp Auto”) and TFσ peaks (“TF Auto”). The top row shows the average spectrograms from all unique events detected by both methods (“Both”) or uniquely identified by a given method (“Only”). The bottom four subpanels show the histograms of event counts for the given methods corresponding to different morphological properties: prominence, duration, central frequency, and bandwidth. Very similar results as those obtained in the second night and reported in Figure 2d are found, further demonstrating the robustness of the identified patterns.

**
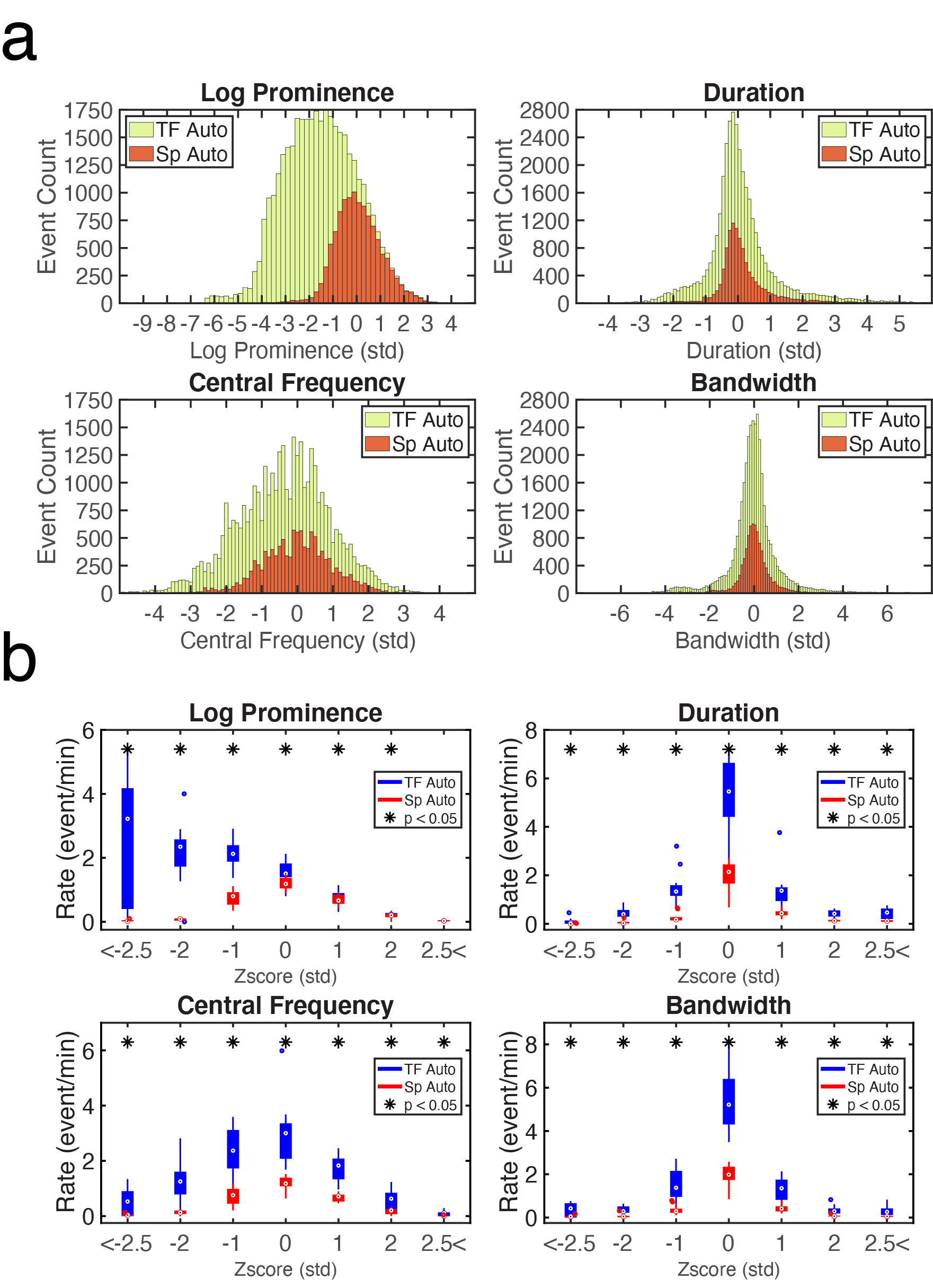
**

Figure S6: Results from the first night replicate the pattern that TFσ peaks outnumber spindles across a wide range of event morphologies. Binned tests were performed for auto-detected spindles (“Sp Auto”) and TFσ peaks (“TF Auto”). (a) shows the distributions of z-scored event properties as in Figure S3a-d. (b) shows boxplots and non-parametric test significant bins on the z-scored event properties. These results replicate the findings found for the second night and hand-scored events that the increased number of TFσ peaks with lower prominence is driven by events with otherwise comparable morphological properties as spindles.

# References

1. Helman A. *The Finest Peaks-Prominence and Other Mountain Measures*. Trafford Publishing; 2005.

2. Pereda E, Gamundi A, Rial R, González J. Non-linear behaviour of human EEG: fractal exponent versus correlation dimension in awake and sleep stages. *Neurosci Lett*. 1998;250(2):91-94.

3. Devuyst S. The DREAMS Databases and Assessment Algorithm [Data set]. http://doi.org/10.5281/zenodo.2650142. Published 2005.

4. Mason DM, Schuenemeyer JH. A modified Kolmogorov-Smirnov test sensitive to tail alternatives. *Ann Stat*. 1983:933-946.
